# Hydrogen Atom Abstraction and Reduction Study of 21-Thiaporphyrin and 21,23-Dithiaporphyrin

**DOI:** 10.3390/molecules29143424

**Published:** 2024-07-22

**Authors:** Xiao-Rui Ren, Kang Xing, Teng Liu, Ronghui Cao, Li-Long Dang, Feng Bai, Peng-Cheng Duan

**Affiliations:** 1 Key Laboratory for Special Functional Materials of Ministry of Education, National and Local Joint Engineering Research Center for High-Efficiency Display and Lighting Technology, School of Nanoscience and Materials Engineering, Henan University, Kaifeng 475004, China; 2 College of Chemistry and Chemical Engineering, Luoyang Normal University, Luoyang 471934, China

**Keywords:** hydrogen atom abstraction, reduction, thiaporphyrins, X-ray diffraction, ZINDO/S calculation

## Abstract

The metal-free porphyrins protonation has gained interest over five decades because its structure modification and hardly monoacid intermediate isolation. Here, upon the hydrogen atom abstraction processes, one step diproptonated H_3_STTP(BF_4_)_2_ (STTP = 5,10,15,20-tetraphenyl-21-thiaporphyrin) (**3**) and stepwise protonated HS_2_TTPSbCl_6_ (**5**) and diprotonated H_2_S_2_TTP(BF_4_)_2_ (**6**) (S_2_TTP = 5,10,15,20-tetraphenyl-21,23-thiaporphyrin) compounds were obtained using HSTTP and S_2_TTP with oxidants. The closed-shell protonated compounds were fully characterized using XRD, UV-vis, IR and NMR spectra. In addition, the reduced 19π compounds [K(2,2,2)]HSTTP (**2**) and [K(2,2,2)]S_2_TTP (**7**) were synthesized by the ligands with reductant KC_8_ in THF solution. These two open-shell compounds were characterized with UV-vis, IR and EPR spectroscopies. The semiempirical ZINDO/S method was employed to analyze the HOMO/LUMO gap lever and identify the electronic transitions of the UV-vis spectra of the closed- and open-shell porphyrin compounds.

## 1. Introduction

Porphyrins, as innocent ligands, have been widely used to mimic biological functions, organometallic, and supramolecular self-assembly [1,2,3]. The pyrrole ring was replaced by one or two thiophene rings in the porphyrin macrocycle ligand, which can effectively reduce the valence of porphyrin for helping low-valent transition complex construction [4,5,6,7] and multiporphyrin assembly [8,9].

**Scheme 1:**
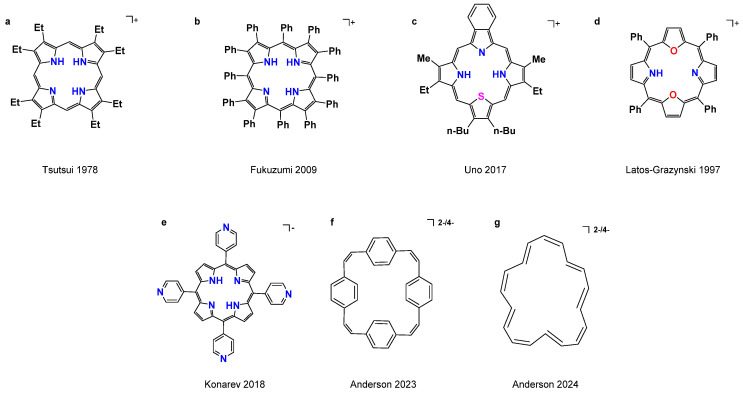
The prominent examples of characterized structures for the mono-protonation porphyrins complexes and for the reduction of macrocyclic hydrocarbons [18,19,24,25,28,29,30].

The hydrogen atom abstraction (HAA) strategy is a fundamental process in redox [10], carbohydrate functionalization [11,12], and catalysis reactions [13,14]. It often reflects the synchronicity of concerted H^+^/e^−^ transfers. Enzymes with various porphyrin analogues (P450, F430 and vitamin B12) performing the HAA catalysis from an alkyl group is an important concept [15]. However, as innocent ligands, the metal-free porphyrins are seldom used for study in the HAA strategy with pairwise H^+^/e^−^ transfers. Detecting and isolating mono-protonated intermediate porphyrins in the protonation process is very difficult. Until now, only a few mono-protonated structurally or spectroscopically characterized porphyrins have been reported (Scheme 1) [16,17,18,19,20,21,22,23,24,25]. The first structure characterized was reported by Takenaka et al. in 1974 using octaethylporphyrin with HI at room temperature (Scheme 1a) [17]. Fukuzumi reported the first monoprotonated porphyrin compound with α,β and meso-substituted (Scheme 1b) [18]. Latos-Grazynski studied the monoprotonation of N_3_S-porphyrin with spectroscopies, and Uno first structurally characterized mono-protonation N_3_S^23^-benzoporphyrin in 2017 (Scheme 1c) [23,24]. In these reported structure characterizations, the strong acids and α, β-substituents porphyrins are essential for synthesized mono-protonated porphyrin complexes. So far, only one structural determination has been reported for the monoprotonation porphyrin with only meso-substituted (Scheme 1d) [25].

The π-conjugated macrocyclic demonstrates unusual global electronic delocalization structures, aromaticity, and optical properties upon reduction by alkali metals [26,27]. Very recently, Anderson et al. reported the anti-aromatic and aromatic cyclophane and [18] annulene through two- or four-electron reduction (Scheme 1f,g) [28,29]. These compounds demonstrate surprising conformation changes and optimization of π-bonding interaction. And the metal-free reduced porphyrite macrocycles were always used to study delocalization and charge disproportionation (Scheme 1e) [30,31].

Before cobalt 21-thiaporphyrin complexes in three different oxidation states and reactivities were explored by us [7], here we were able to undergo one-step HAA strategy results in diprotonation 21-thiaporphyrin and step-wise HAA strategy results in mono- and di-protonation 21,23-thiaporphyrin. The protonated porphyrins were characterized with X-ray crystallography and spectroscopies. In addition, the two porphyrin 19π potassium salts were isolated and fully characterized. To the best of our knowledge, this is the first mono- and di-protonated metal-free porphyrins by oxidants through the HAA process.

## 2. Results and Discussion

**Figure 1:**
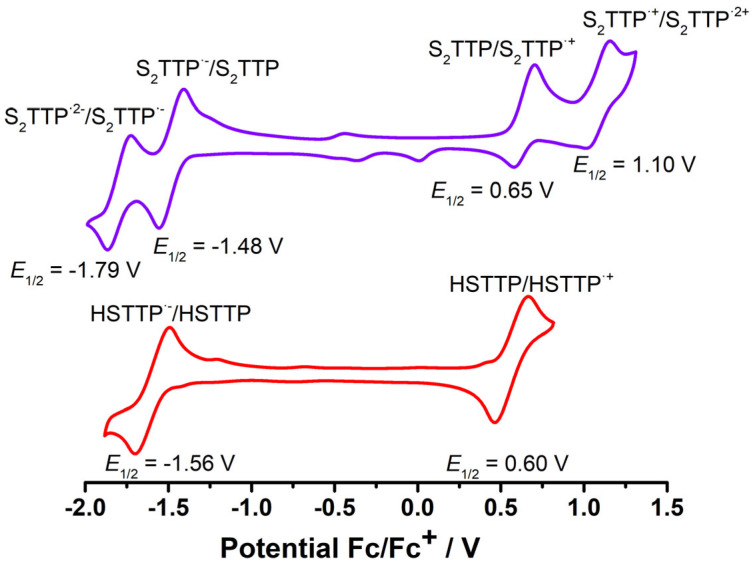
Cyclic voltammogram (CV) of HSTTP (red) and S_2_TTP (violet) in DCM solution under N_2_ atmosphere.

The electrochemistry of HSTTP and S_2_TTP were probed using a cyclic voltammetry (CV) method in DCM solution under an N_2_ atmosphere (Figure 1). The two reversible redox potentials at *E*_1/2_ = −1.56 V and +0.6 V (vs Fc/Fc^+^) were observed for HSTTP, which were assigned to HSTTP^·−^/HSTTP and HSTTP/HSTTP^+^ processes (Figure S4). Two reversible reduction and two reversible oxidation potentials at *E*_1/2_ = −1.56 and −1.48 V and 0.65 and 1.10 V (vs Fc/Fc^+^), respectively, were seen for S_2_TTP, which were assigned to S_2_TTP^2−^/S_2_TTP^−^, S_2_TTP^−^/S_2_TTP, S_2_TTP/S_2_TTP^+^, and STTP^+^/HSTTP^2+^. However, the redox process for S_2_TTP^2−^/S_2_TTP^−^ and STTP^+^/HSTTP^2+^ are irreversible at a high scan rate (Figure S18).

### 2.1. H-Atoms Abstraction and Reduction in 21-Thiaporphyrin

#### 2.1.1. Syntheses

**Scheme 2:**
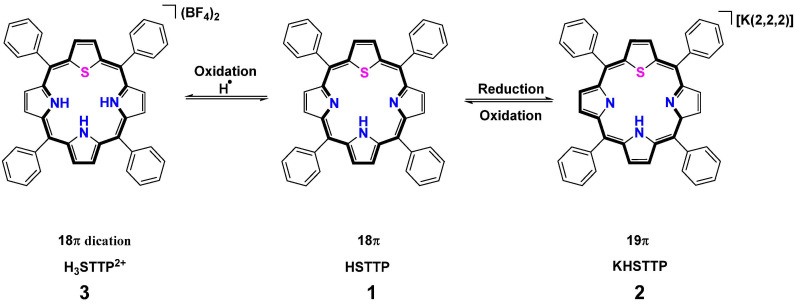
Synthetic transformations of the HSTTP (**1**) to 19π salt (**2**) and 18π dication (**3**).

**Figure 2:**
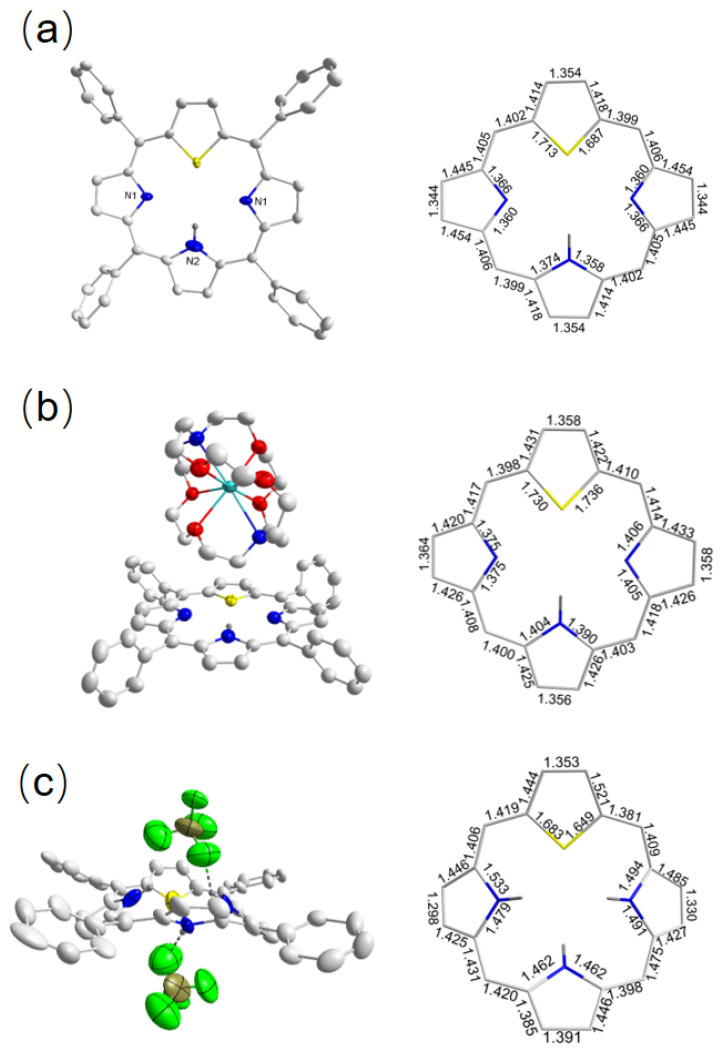
Left: Solid-state structure of the HSTTP **1** (**a**), potassium salt **2** (**b**), and 18π diacids **3** (**c**); right: bond lengths for the porphyrin cores of HSTTP **1** (**a**), potassium salt **2** (**b**), and 18π diacids **3** (**c**). The refined positions of the hydrogen atoms bonded to the pyrrole nitrogens are shown.

Reaction of HSTTP with excess KC_8_ in the presence of [2,2,2]*C*ryptand in THF solution results in the color changing from brown-red to cyan immediately. A structurally identical sample of 19π salt [Na(2,2,2)]HSTTP could be generated from the parent ligand HSTTP with Na/NaCl in THF, and petrol diamond-shaped crystals were obtained from pentane and slowly diffused into a THF/CH_3_CN mixture overnight at rt. The solid structure of sodium salt **2** is shown in Figure 2b. Using the NOBF_4_ to oxidize HSTTP in a DCM solution resulting in a color change from red the deep green, a 18π dication complex was generated instead of a 17π complex or 18π cation intermediates. The proton for 18π dication salt may generated by H-atom abstraction from a solvent. An alternative way to synthesize this complex was using ligand 1 with excess HBF_4_ in a DCM solution. Black-green block crystals of [H_3_STTP](BF_4_)_2_ (**3**) were obtained by layering a CH_3_CN mixture with Et_2_O at rt in two days (Scheme 2).

#### 2.1.2. Crystallographic Details

New 19π sodium salt **2** and 18π diacids **3** complexes were characterized using single X-ray crystallography (Figure 2). Compared to the free ligand **1**, no obviously changed bond lengths and angles were viewed in 19π complex **2** (Figure 2a,b). And the thiaporphyrin macrocycle ring was almost planar with slightly tile-up sulphur atom in thiophene by 0.12 Å. In the diacids complex **3**, the non-planar porphyrin macrocycle in a saddle shape [2] was observed in the solid-state structure. The pyrrole rings and thiophene ring were disoriented with 33.40(4)° (N1), 34.88(4)° (N2), 32.81(4)° (N3), and 27.72(3)° (S1), respectively, from the 21-thiaporphyrin macrocycle plane. These values about disoriented pyrrole angles are much larger than the 2H^+^·N_3_S-BCOD thiaporphyrin (15.8°, 13.9° and 19.7°) [24]. Two H-bonding with 1.887(2) and 2.337(2) Å were found in the new proton and BF4 anion partners. The two BF_4_ anions were located in the top and bottom of thiaporphyrin saddle. The C-N bonds of the pyrrole ring in the opposite thiophene ring were increased in complexes **1**–**3** from 1.358(2) to 1.462(2) Å (Figure 2, right). And the C-S bonds in complexes **1**–**3** were irregularly changed from 1.687 Å (**1**) to 1.736 Å (**2**) and then decreased to 1.649 Å (**3**). The N-N distances in the opposite pyrrole rings were 4.40(3) (**1**), 4.40(1) (**2**) and 4.20(2) (**3**), respectively. The non-bonding N···N distance was slightly shortened (4.40 Å in **1**, 4.29 Å in **2** and 4.21 Å in **3**), and the non-boding N···S distance became longer (3.69 Å in **1**, 3.77 Å in **2** and 3.92 Å in **3**).

#### 2.1.3. Spectra Characterization

**Figure 3:**
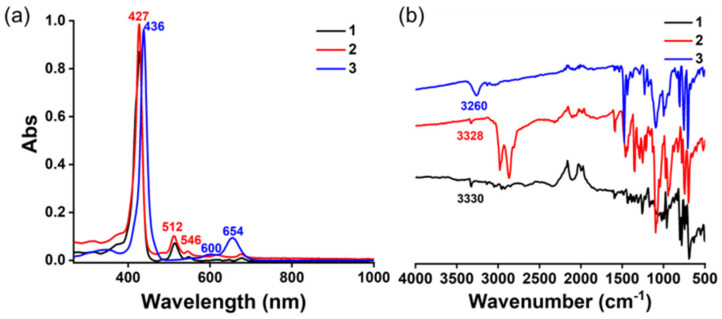
(**a**) UV−Vis spectra of HSTTP **1** (black line, in DCM), sodium salt **2** (red line, in THF), and 18π diacids **3** (blue line, in CH_3_CN) were measured in the solution state; (**b**) ATR-IR spectrum of HSTTP **1**, potassium salt **2**, and 18π diacids **3** in crystalline material.

UV-Vis spectra of free-base HSTTP **1**, potassium salt **2**, and diacids **3** are shown in Figure 3a. The HSTTP **1** and 19π salt **2** have almost the same Soret bands at 375 and 427 nm, and several Q-bands were at 512, 546 and 616 nm. Compared with the other 19π porphyrins salts [30,31], no NIR bands around 900 nm were observed in our system. In diacids **3**, the Soret band is weakly blue shifted compared with those in the spectrum of HSTTP **1** and salt **2** and appear at 436 nm. New low-energy bands were observed in the spectrum of diacids 3 at 600 and 654 nm. ATR-IR spectra of HSTTP **1**, salt **2**, and diacids **3** are shown in Figure 3b. In ligand HSTTP and potassium salt **2**, the bands characteristic of the NH vibrations are observed at 3330 cm^−1^ and 3328 cm^−1^, respectively. The band is shifted to a lower value (3260 cm^−1^) at the formation of diacids **3**. The ^1^H NMR spectrum of diacids **3** in CDCl_3_ shows two signals at −1.95 and −2.56 ppm for the NH groups (Figure S12). However, these NH signals were not observed with the CD_3_CN used for NMR spectrum (Figure S10). The X-band EPR spectrum of sodium salt **2** in THF solution recorded at rt shows an organic radical signal with the *g* = 2.003 (Figure S5). It is very close to the free radical (*g* = 2.0023) and other 19π porphyrins salts [30,31].

### 2.2. Stepwise H-Atom Abstraction and Reduction in 21,23-Thiaporphyrin

#### 2.2.1. Syntheses

**Scheme 3:**
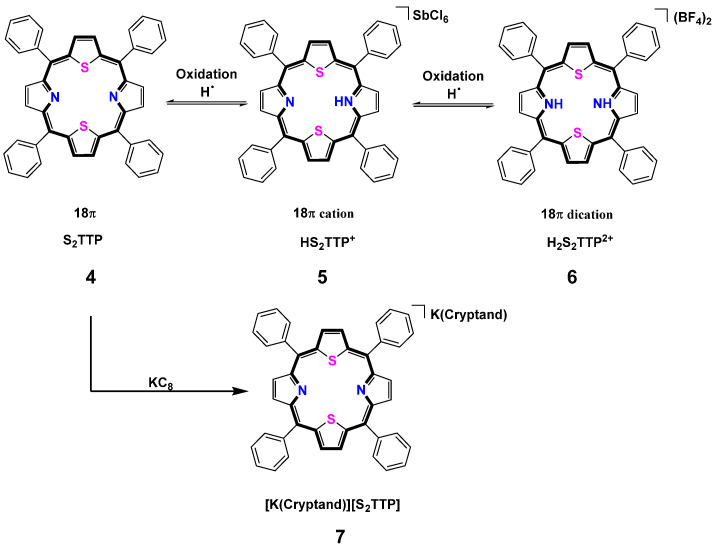
Synthetic transformations of the S_2_TTP ligand to 18π monoacid **5**, 18π diacids **6** and 19π **7**.

Using one equivalent magic blue (Tris(4-bromophenyl)ammoniumyl hexachloroantimonte) or two equivalents NOBF_4_ with S_2_TTP in a CHCl_3_ solution resulting in an immediate color change from brown-red to grass green and green precipitates were generated (Scheme 3). The monoacid **5** and diacids compound **6**, which had poor solubility in CHCl_3_ and methanol and totally deprotonated in a CH_3_CN solution. Green block crystals of monoprotonated complex HS_2_TTP(SbCl_6_) (**5**) were grown from hexane and slowly diffused into the THF/CHCl_3_ mixture solution in a week. And the blue needle-shaped crystals of diacids complex **6** for X-ray diffraction were obtained from Et_2_O diffusion into a CHCl_3_/CH_3_CN mixture solution in the presence of HBF_4_·Et_2_O drops in three days. The reaction of S_2_TTP with KC_8_ in the presence of [2,2,2]cryptand in the THF solution results in a color change from brown-red to greenish. The solubility of the 19π potassium salt is also poor in THF and CH_3_CN. Yellowish diamond-shaped crystals of [K(2,2,2)]S_2_TTP (**7**) were obtained from hexane diffusion into the CH_3_CN/THF mixture solution.

#### 2.2.2. Crystallographic Details

**Figure 4:**
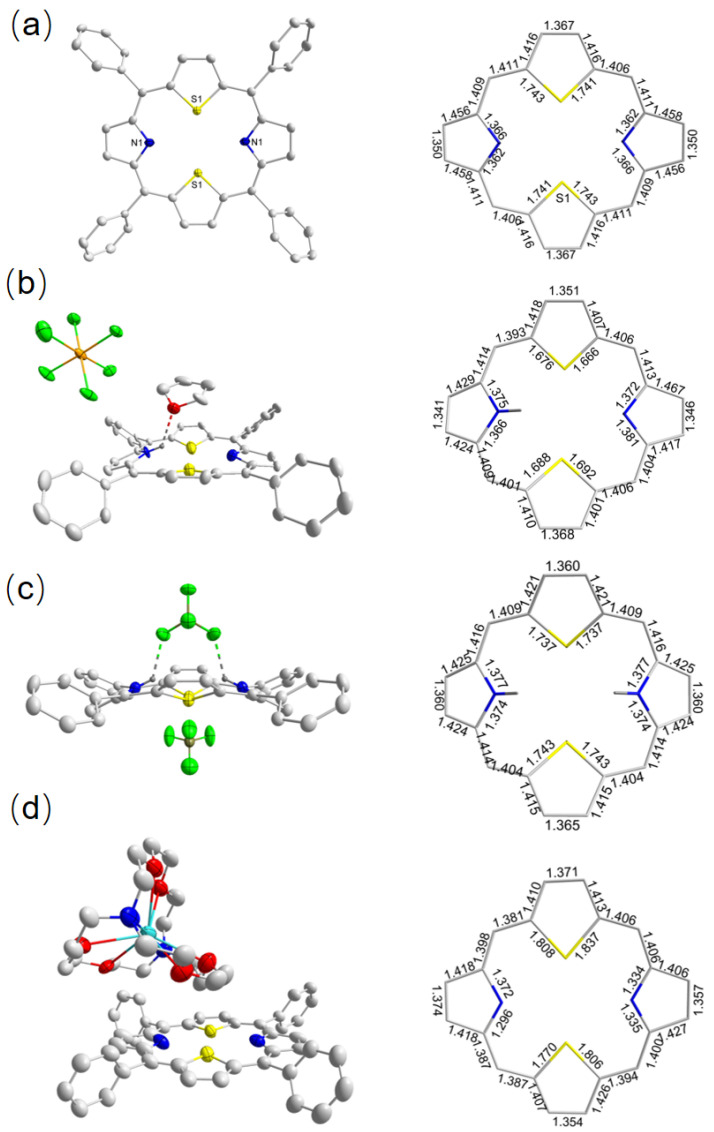
Left: Solid-state structure of the S_2_TTP **4** (**a**), 18π acid **5** (**b**), 18π diacids **6** (**c**) and 19π potassium salt (**d**); Right: Bond lengths for the porphyrin cores of S_2_TTP **4** (**a**), 18π acid **5** (**b**), 18π diacids **6** (**c**) and 19π potassium salt (**d**); The refined positions of the hydrogen atoms bonded to the pyrrole nitrogens are shown.

The monoacid **5**, diacids **6**, and 19π **7** complexes were characterized by single X-ray crystallography (Figure 4b–d). The two acid complexes were both adopted saddle shaped. In complex 5, the pyrrole rings and thiophene rings were disoriented with 21.28(2)° (N1), 9.31(2)° (N2), 16.79(1)° (S1), and 15.93(1)° (S2), respectively, from the 21,23-thiaporphyrin macrocycle plane. A similar monoprotonated compound was observed in the [(O_2_TPPH)_2_][NiCl_4_] compound [25]. And in complex **6**, the disoriented angles were 26.75(2)° (N1 and N1’), 22.20(6)° (S1), and 29.77(2)° (S2), respectively. In complex **5**, an H-bonding with 2.142(5) Å was observed from the NH and THF solvent. The SbCl_6_ anion was located in the periphery of 21,23-thiaporphyrin. In complex **6**, however, two H-bondings with 2.063(2) Å were found in the NH protons and one BF_4_ anion partner. The BF_4_ anion in the outer ring of 21,23-thiaporphyrin linked four porphyrins through H−bonding (2.514–2.764 Å), resulting in a 3D network arrangement. (Figure S36) The C-N bonds of pyrrole rings increased in complexes **4**–**6** from 1.36(2) to 1.38(2) Å (Figure 4, bottom). And the C-S bonds in complexes **4**–**6** were irregularly changed from 1.74 Å (**4**) to 1.68 Å (**5**) and then increased to 1.74 Å (**6**). The quality and resolution of the complex **7** was limited, because the compound contained cryptand and solvents; the structure of [K(Cryptand)]S_2_TTP still provided important information for comparison. Two similar crystallographically molecules of complex **7** were found in the asymmetric, and only one is shown. The reduced 19π complex **7** was like the parent ligand S_2_TTP, again without significant changes (Figure 4d). The C-S bonds were the largest bonds compared to the complexes **4**–**6**. In addition, the N···N distances in the opposite pyrrole rings were 4.40(3) (1), 4.29(9) (2) and 4.21(2) (3), respectively. The selected metric parameters for the tilted angles of five-membered ring, C-N-C, C-S-C angles, and the average length of the C-S and C-N bonds are listed in the Table 1. More detailed bond lengths and angles can be found in the Supplementary Information.

#### 2.2.3. Spectra Characterization

**Figure 5:**
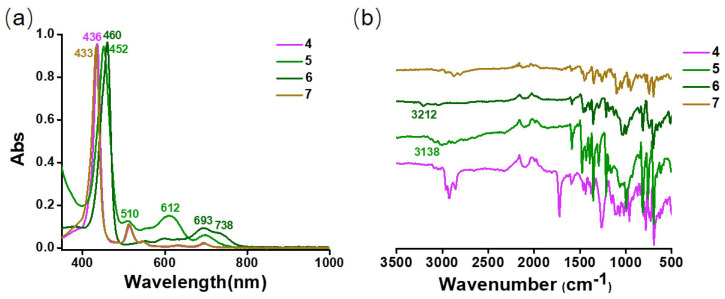
(**a**) UV−Vis spectra of S_2_TTP **4** (Vilot in DCM), monoacid **5**, and diacids **6** (green line, in CHCl_3_) and **7** (orange line, in THF) were measured in solution state; (**b**) ATR-IR spectrum of S_2_TTP **4** (Vilot), 18π monoacid **5** (green), diacids **6** (deep green), and 19π potassium salt **7** (orange) in crystalline material.

The UV-vis spectra of S_2_TTP ligand **4**, monoacid **5**, diacids **6**, and 19π compound **7** are shown in Figure 5a. Similar absorption at Sort and Q bands were observed in the parent ligand S_2_TTP and open-shell compound **7**. In 19π compound **7**, still no NIR bands were viewed in the solution state. In monoacid compound **5**, the band at 452 nm was blue-shifted compared to the parent S_2_TTP ligand. Two low-energy bands at 612 and 698 nm were observed in the spectrum. In the diprotonated compound **7**, a closed-NIR band at 738 nm was viewed compared to all the parent, reduced, and monoprotoned compounds. The IR spectra show the band at 3138 cm^−1^ for **5** and 3212 cm^−1^ for **6**, which was assigned to the NH stretching vibration of the protonation of 21,23-thiaporphyrin (Figure 5b). Since the monoacid **5** and diacids **6** compounds had bad solubility in the CHCl_3_ solution, no signals assigned to the NH peak were observed in the ^1^H NMR spectrum (Figure S24). In monoacid compound **5**, a *C*_2V_ symmetric was observed at room temperature, indicating that the proton in the NH unit had a rapid interconvert at the NMR time scale. The X-band EPR spectrum of potassium salt **7** in 2-MeTHF solution recorded at 106 K shows an organic radical signal with the *g* = 2.003, which is the same as compound **2** (Figure S29).

### 2.3. ZINDO/S Calculation

**Figure 6:**
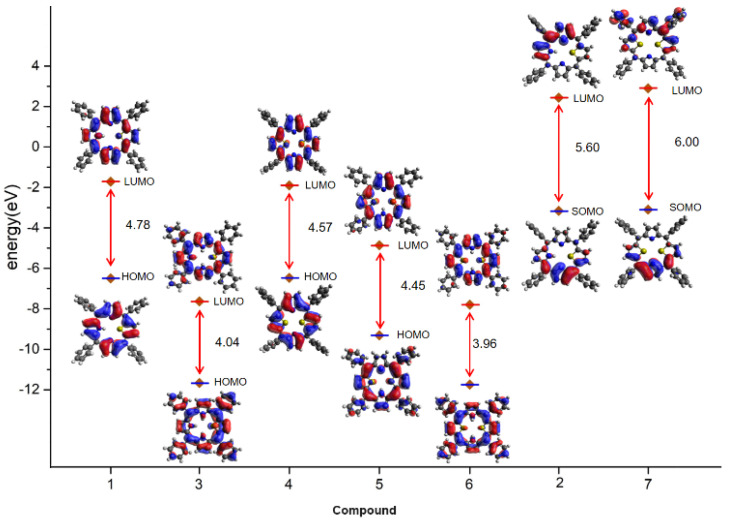
Illustration of the HOMOs/SOMOs and LUMOs for compounds **1**–**7** from ZINDO/S calculation. The values were obtained from the ZINDO calculations for the X-ray geometries.

The electronic structures and transition energies for the X-ray geometries of the 21- and 21,23-thiaporphyrins compounds **1**–**7** were studied using the ZINDO/S method with ORCA program in version 4.2.1 [32,33,34]. The HOMO(SOMO)/LUMO energy gaps for compounds **1**–**7** are shown in Figure 6 and Figures S38–S44. In compounds **1** and **4**, the energy gap difference is 4.78 ev and 4.57 ev, respectively, and it is inferred that the electrons are more easily transferred in compound 4. After protonation, the energy gap differences decreased with 4.04 eV (**3**), 4.45 eV (**5**), and 3.96 eV (**6**), respectively. This indicates that the electrons transit more easily from HOMO to LUMO in compounds **3**, **5**, and **6**, and the corresponding absorptions in acid **3**, **5**, and **6** are more red-shifted. In the reduced salts **2** and **7**, the LUMO lever is both positive (>0), and the energy gap between SOMO and LUMO are 5.6 eV and 6.0 eV, respectively. These results combined with the experimental process indicate that the open-shell compound **7** cannot be reduced anymore, even though the CV measurement demonstrates that compound **7** has the second reversible reduction potential (Figure 1).

The UV-vis absorption spectra in closed-shell porphyrin derivatives can be assigned S0→S2 transition around 350–550 nm and S0→S1 transition around 600–1000 nm. As can be seen from Figures S43–S49, the calculated UV-vis absorption spectra are in close agreement with the observed spectra pattern in compounds **1**–**7**. In the closed-shell compound, the four orbitals labeled as H, H−1, L, and L+1 play an important role in the energy electronic transitions (Table S2). However, in the open-shell compound **2** and **7**, the orbits about S−7, S−5, and L+5 et al. also have some contribution in the energy electronic transitions.

## 3. Materials and Methods

### 3.1. Instruments

Schlenk techniques and a nitrogen atmospheric drybox (Vigor Technology Inc., Vancouver, BC, Canada) were used for handling air-sensitive compounds. ^1^H NMR spectra were measured on a Bruker Avance spectrometer (500 MHz, Billerica, MA, USA). Chemical shifts are expressed in parts per million relative to residual CHCl_3_ (*δ*H = 7.26 ppm) and CD_3_CN (*δ*H = 1.94 ppm). IR spectra of crystalline samples were measured with a Cary 630 FTIR spectrometer equipped with a DialPath and Diamond ATR accessory (Agilent, Santa Clara, CA, USA) placed in a glovebox (N_2_ atmosphere, Vigor Technology Inc., Vancouver, Canada). IR bands were labeled according to their relative intensities with vs. (very strong), s (strong), m (medium), w (weak), and very weak (vw). UV−vis spectra were recorded on an Agilent Cary 60 (Santa Clara, CA, USA). Compounds **2** and **7** were prepared in a glovebox and transferred out of the glovebox prior to the measurement. Cyclic voltammetry (CV) experiments were performed with an Interface 1000B potentiostat (Vesion 4.5, Gamry Instruments, Warminster, PA, USA) using a three-electrode setup consisting of a glassy carbon working electrode, a platinum wire counter electrode, and an Ag reference electrode and were analyzed using Gamry Framework software (Version 7.8.6). CV experiments were performed in deoxygenated DCM containing ^n^Bu_4_PF_6_ (0.1 M) as the supporting electrolyte; ferrocene was used as an internal standard. ESI mass spectra were recorded on a Bruker HCT ultra spectrometer (Billerica, MA, USA).

### 3.2. Materials

Solvents were dried by standard methods and freshly distilled prior to use. Dichloromethane and chloroform were dried with calcium hydride and distilled under nitrogen. THF, hexane, and pentane were distilled under nitrogen in the presence of sodium chips using benzophenone ketyl as an indicator. Dried solvents, which were transferred into round-bottom flasks, bubbled with nitrogen for at least 10 min to remove residual dioxygen and then sealed with a J-Young cap and were stored in the nitrogen atmosphere drybox prior to use. Pyrrole was freshly distilled under nitrogen from calcium hydride prior to use. Other starting materials were obtained commercially and used directly without further purification. Silica gel (100–200 mesh) or neutral alumina was used for column chromatography. The starting 5,10,15,20-tetraphenyl-21-thiaporphyrin (HSTTP) and 5,10,15,20-tetraphenyl-21,23-thiaporphyrin (S_2_TTP) [35], Na/NaCl (5%) [36] were prepared according to the literature with modification.

### 3.3. EPR Spectroscopy

Continuous-wave (cw) X-band EPR measurements were performed on a Bruker A200 spectrometer equipped with a high sensitivity cavity (ER4119HS) in conjunction with microwave bridge Bruker A40X (Billerica, MA, USA). For variable temperature control, cryostat (A4131VT) was employed. EPR simulations have been carried out with esim due to Dr. Eckhard Bill at the Max-Planck-Institut für Chemische Energiekonversion [37] and Easyspin program [38].

### 3.4. Single-Crystal X-ray Structure Determinations

Crystal data and details of the data collections are given in Table S1. X-ray data were collected on a STOE IPDS II diffractometer (Darmstadt, Germany) or Rigaku Oxford diffractometer (graphite monochromated Mo−Kα radiation, λ = 0.71073 Å, Tokyo, Japan) by use of scans at 150 K or 100 K. The structures were solved using SHELXT [39] and refined on *F*^2^ using all reflections with SHELXL2014/16 [40] interfaced with Olex2 [41]. All Non-hydrogen atoms were refined anisotropically. Most hydrogen atoms were placed in calculated positions and assigned to an isotropic displacement parameter of 1.2/1.5 Ueq(C). All unit cells contained highly disordered solvent molecules for which no satisfactory model for a disorder could be found. The solvent contribution to the structure factors was calculated with PLATON SQUEEZE [42]. ISOR, SADI, SAME, and EADP restraints were applied to model the disorder. The CCDC deposition numbers 2348693–2348699 contain the supplementary crystallographic data. This data can be obtained free of charge via The Cambridge Crystallography Data Centre.

### 3.5. Synthesis of [Na(Cryptand)](HSTTP) (***2***)

Na/NaCl (5%) (22 mg, 0.05 mmol, 1 equiv) was added to a solution of HSTTP (30 mg, 0.05 mmol, 1 equiv) in THF in the presence of 1 equiv of [2.2.2]cryptand. The reaction mixture became green within 30 min and was stirred overnight at room temperature to yield a cyan solution. The solvent was removed under a vacuum. The residue was then dissolved in THF, and the NaCl and graphite were removed by filtration through celite. After layering with hexane, the product was obtained as blue-plate crystals after overnight (yield: 57%). ATR-IR (ν/cm^−1^) = 3328 (w), 2977 (s), 2869 (s), 1588 (m), 1457 (m), 1437 (m), 1354 (s), 1291 (m), 1252 (m), 1212 (m), 1130 (w), 1096 (vs), 1042 (s), 979 (m), 945 (s), 779 (m), 746 (s), 702 (s), and 521 (w). UV-vis (THF) = 427, 512, 546, 616, and 675 nm. EPR(THF) *g*_x_ = 2.007, *g*_y_ = 2.003, and *g*_z_ = 1.999.

### 3.6. Synthesis of [H_3_STTP](BF_4_)_2_ (***3***)

HSTTP (100 mg, 0.158 mmol, 1 equiv) and NOBF_4_ (18.5 mg, 0.138 mmol, 1 equiv) were dissolved in 3 mL of DCM, resulting in a color change from brown-red to black-green immediately. After stirring overnight at rt, the residual was filtered through a patch of celite. Green-block suitable black-block crystals for X-ray diffraction were obtained by layering Et2O on a solution of 1 in CH_3_CN (Yield: 70%). ^1^H NMR (500 MHz, CDCl_3_, 298 K) = 9.67 (s, 2H), 8.95 (d, J = 4 Hz, 2H), 8.80 (d, J = 4 Hz, 2H), 8.67 (d, J = 8 Hz, 6H), 8.61 (d, J = 8 Hz, 4H), 8.10 (t, J = 8 Hz, 4H), 8.02 (m, 8H), −1.96 (s, 1H, NH), and −2.50 (s, 2H, NH). ^19^F NMR = −155.89, −155.94. ATR-IR (ν/cm^−1^) = 3260 (m), 1592 (w), 1536 (w), 1475 (vs), 1438 (m), 1377 (w), 1290 (w), 1230 (m), 1094 (s), 999 (s), 806 (s), 752 (vs), and 708 (vs). UV-vis (CH_2_Cl_2_) = 436, 600, and 654 nm.

### 3.7. Synthesis of [HS_2_TTP]SbCl_6_ (***5***)

S_2_TTP (65 mg, 0.10 mmol, 1 equiv) and Magic Blue (52 mg, 0.1 mmol, 1 equiv) were dissolved in 10 mL of DCM, resulting in a color change from brown-red to black-green immediately. After stirring overnight at rt, the residual was filtered through a patch of celite. Green-block suitable black-block crystals for X-ray diffraction were obtained by layering Et_2_O on a solution of 5 in CHCl_3_/THF (Yield: 62%). ^1^H NMR (500 MHz, CDCl_3_, 298 K) = 10.0 (s, 4H), 8.95 (s, 4H), 8.44 (s, 8H), and 7.96 (s, 12H). ATR-IR (ν/cm^−1^) = 3138 (w), 3084 (w), 2998 (w), 2908 (w), 2868 (w), 1587 (m), 1474 (s), 1429 (m), 1389 (m), 1355 (s), 1298 (m), 1218 (s), 1059 (m), 998 (vs), 816 (vs), 760 (s), 697 (vs), and 617 (m). UV-vis (CHCl_3_) = 452, 510, 612, and 698 nm.

### 3.8. Synthesis of [H_2_S_2_TTP](BF_4_)_2_ (***6***)

S_2_TTP (33 mg, 0.05 mmol, 1 equiv) and NOBF_4_ (12 mg, 0.1 mmol, 1 equiv) were dissolved in 3 mL of CHCl_3,_ resulting in a color change from brown-red to green in 5 min. After stirring overnight at rt, the residual was filtered through a patch of celite. Green-block suitable black-block crystals for X-ray diffraction were obtained by layering Et2O on a solution of 6 in CHCl_3_/CH_3_CN mixture with drops of HBF_4_ (Yield: 60%). ^1^H NMR (500 MHz, CDCl_3_, 298 K) = 9.99 (s, 4H), 8.87 (s, 4H), 8.43 (s, 8H), and 7.94 (s, 12H). ^19^F NMR (471 MHz, CDCl3, 298 K) = −155.67, −155.72. ATR-IR (ν/cm^−1^) = 3212 (w), 3045 (w), 1587 (m), 1463 (m), 1440 (m), 1400 (m), 1355 (s), 1292 (m), 1213 (m), 1185 (w), 1122 (m), 1032 (s), 998 (s), 816 (s), 742 (m), 697 (vs), 657 (m), 617 (m), and 516 (m). UV-vis (CHCl_3_) = 460, 552, 598, 693, and 738 nm.

### 3.9. Synthesis of [K(Cryptand)](S_2_TTP) (***7***)

KC_8_ (4.2 mg, 0.03 mmol, 1 equiv) was added to a solution of S2TTP (20 mg, 0.03 mmol, 1 equiv) in THF in the presence of 1 equiv of (2,2,2)cryptand. The reaction mixture was stirred overnight at room temperature to yield a chartreuse solution. The solvent was removed under a vacuum. The residue was then dissolved in THF, and graphite was removed by filtration through celite. After layering with hexane, the product was obtained as brown-plate crystals after overnight (yield: 65%). ATR-IR (ν/cm^−1^) = 3051 (w), 2964 (w), 2878 (m), 2809 (m), 1596 (m), 1479 (m), 1449 (s), 1350 (s), 1290 (m), 1260 (s), 1215 (m), 1135 (m), 1104 (vs), 1044 (s), 972 (s), 945 (vs), 870 (m), 829 (m), 783 (m), 745 (vs), 700 (vs), 647 (w), 628 (w), and 602 (w). UV-vis (THF) = 433, 513, 545, 630, and 694 nm. EPR(2-MeTHF) *g*_x_ = 2.006, *g*_y_ = 2.003, and *g*_z_ = 2.001.

## 4. Conclusions

In summary, the hydrogen atom abstraction and reduction behaviors were explored in the 21-/21,23-thiaporphyrin ligand. the H^+^/e^+^-coupled transfer resulting in the monoprotonated and diprotonated thiaporphyrin complexes were isolated and fully characterized. Two newly reduced open-shell 21-/21,23-thiaporphyrin metal salts are paramagnetic with S = 1/2 spin states. In addition, the protonation properties and antiaroma with a strong electronic group in α,β-substituents of thiaporphyrin were explored in our group.

## Figures and Tables

**Table 1:** Lengths and angles for the **1**, **2**, **3**, **4**, **5**, **6** and **7**.

		1	2	3	4	5	6	7
Angle (°)	Tilt ^a^	S1 4.62(5) N1 11.39(6) N2 4.12(6)	S1 4.75(8) N1 2.39(1) N2 4.07(1) N3 4.33(1)	S1 27.72(3) N1 33.40(4) N2 34.88(4) N3 32.81(4)	S1 4.98(4) N1 12.99(6)	S1 16.79(1) S2 15.93(1) N1 21.28(2) N2 9.31(2)	S1 22.20(6) S2 29.77(2) N1 26.75(2)	S1 1.780(2) S2 3.350(2) N1 3.921(2) N2 2.391(3)
	C-N-C	105.91(1) 120.52(5)	110.63(5) 111.04(5) 111.81(5)	99.177(1) 102.52(1) 100.92(1)	106.32(2)	109.81(5) 104.72(5)	110.39(3)	119.70(2) 121.13(2)
	C-S-C	88.46(1)	85.71(2)	93.00(8)	91.98(1)	91.98(4) 92.60(4)	93.06(2) 92.58(2)	84.15(5) 82.61(4)
Length (Å) ^b^		1.70 1.36	1.73 1.38	1.68 1.48	1.74 1.36	1.68 1.37	1.74 1.38	1.81 1.33
Nonbonded Length(Å) N-NN(S)-S		4.40(3) 3.69(6)	4.29(9) 3.77(7)	4.21(2) 3.92(1)	4.64(3) 3.06(7)	4.51(4) 3.32(4)	4.81(4) 3.31(2)	4.63(2) 3.06(7)

^a^ The tilted angle of the five-membered ring toward the mean plane of 24 porphyrinoid atoms. ^b^ The average length of the C-S or C-N bonds.

## Data Availability

Data are contained within the article and Supplementary Materials.

## Supplementary Materials

The following supporting information can be downloaded at: https://www.mdpi.com/article/10.3390/molecules29143424/s1.
