# Supplementary material for: Hydrogen Atom Abstraction and Reduction Study of 21-Thiaporphyrin and 21,23-Dithiaporphyrin
Source: Molecules. 2024 Jul 22;29(14):3424. doi: 10.3390/molecules29143424 (PMC11279893; doi:10.3390/molecules29143424)
Supplement: Supplementary file 1 [file molecules-29-03424-s001.zip › molecules-3080266-supplementary.pdf]

Supporting Information

Hydrogen Atom Abstraction and Reduction Study of 21-Thiaporphyrin and 21,23-Dithiaporphyrin

Xiao-Rui Ren,[a] Kang Xing,[a] Teng Liu,[a] Ronghui Cao,[b] Li-Long Dang,[b*] Feng Bai,[a*] Peng-Cheng Duan[a*]

a. Key Laboratory for Special Functional Materials of Ministry of Education, National and Local Joint Engineering Research Center for High-Efficiency Display and Lighting Technology, School of Nanoscience and Materials Engineering, Henan University, Kaifeng,475004, China;

b. College of Chemistry and Chemical Engineering, Luoyang Normal University, Luoyang, 471934, China.

* To whom correspondence should be addressed. Email: pduan@henu.edu.cn; baifengsun@126.com; danglilong8@163.com

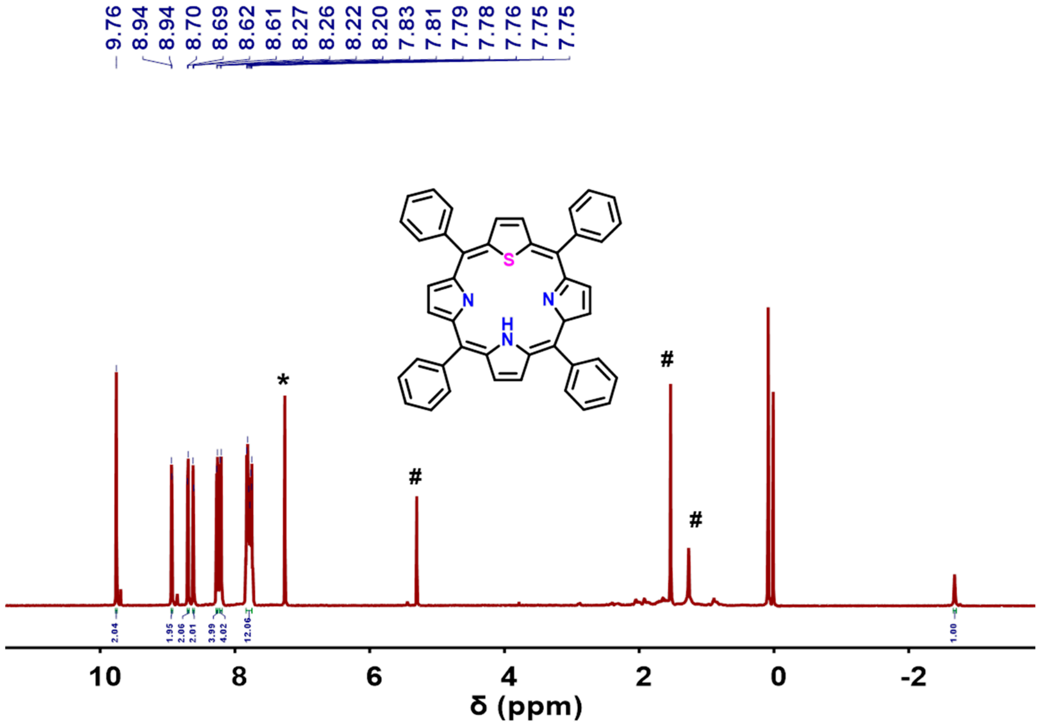

Figure S1: ^{1}H NMR spectrum (500 MHz) of **1** in $CDCl_3$ at 298 K. Solvent signal are marked with an asterisk (*). Other signals are marked with an asterisk (#).

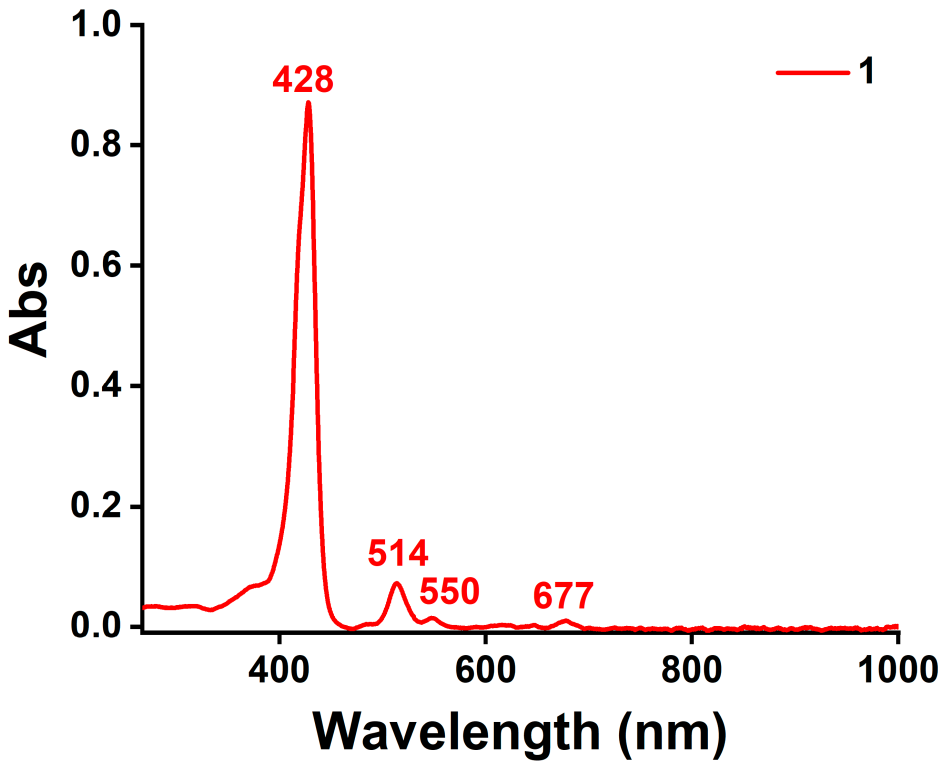

Figure S2: UV-vis absorption spectra of **1** in DCM solution.

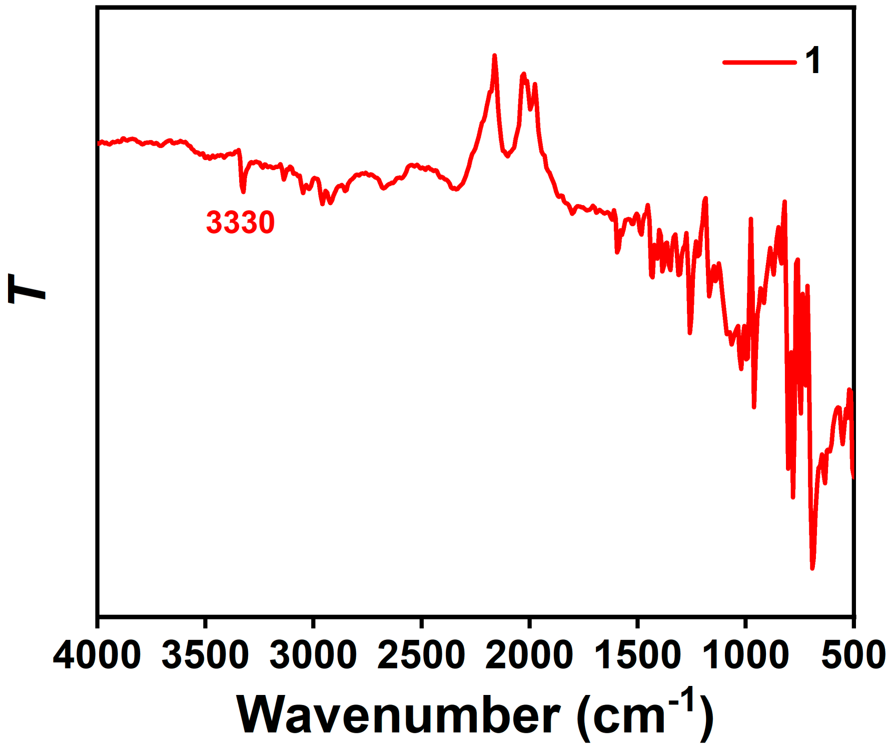

Figure S3: ATR-IR spectrum of **1** in crystalline material.

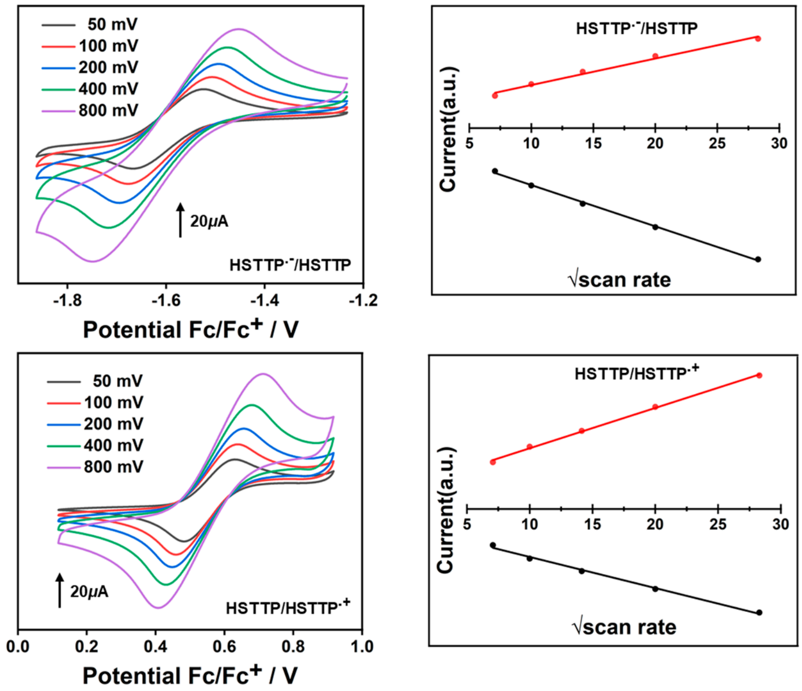

Figure S4: Left Cyclic voltammograms of **1** in DCM (0.1 M $^{n}Bu_4NPF_6$) at different scan rates. (top) $HSTTP^-$/HSTTP redox couple; (bottom) HSTTP/$HSTTP^+$ redox couple. Right: Linear dependence of the current Ifp of the forward peak on the square root of the scan rate. (top) $HSTTP^-$/HSTTP redox couple; (bottom) HSTTP/$HSTTP^+$ redox couple

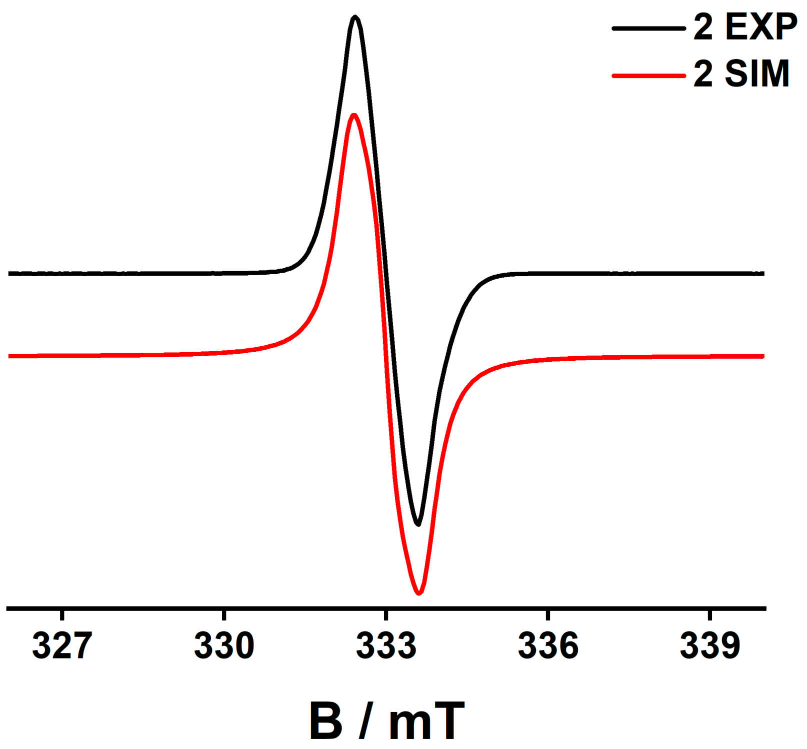

Figure S5: X-band EPR spectrum record at 9.34 GHz, microwave power 1 mW of **2** in THF solution at 293K. The red trace show the simulation with g_{ave} = 2.003.

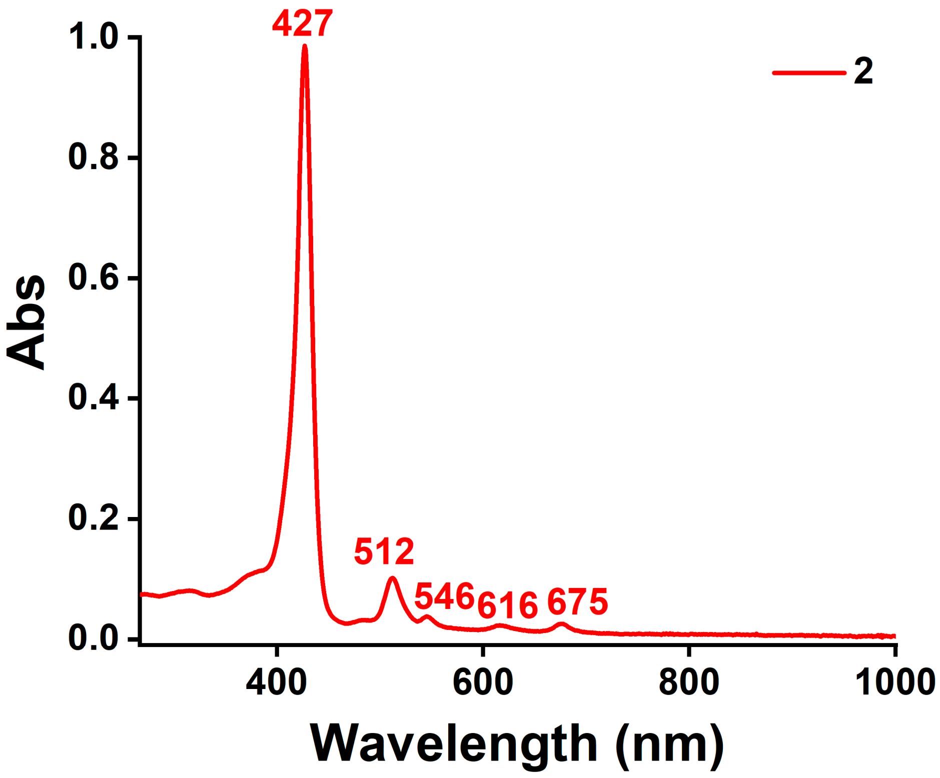

Figure S6: UV-vis absorption spectra of **2** in THF solution.

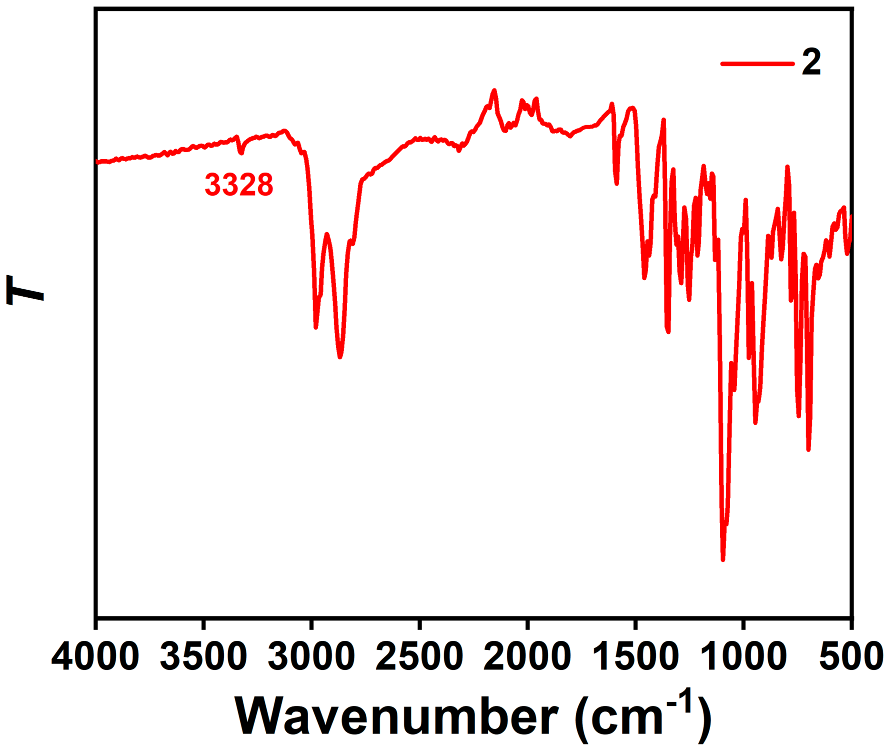

Figure S7: ATR-IR spectrum of **2** in crystalline material.

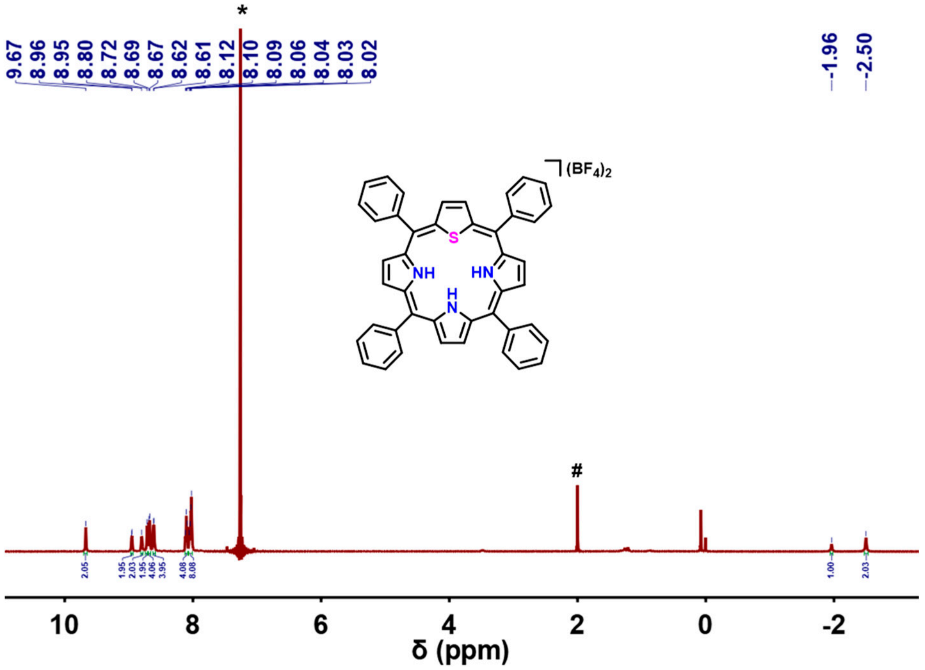

Figure S8: ^{1}H NMR spectrum (500 MHz) of **3** in $CDCl_3$ at 298 K. Solvent signals are marked with an asterisk (*). Other signals are marked with an asterisk (#).

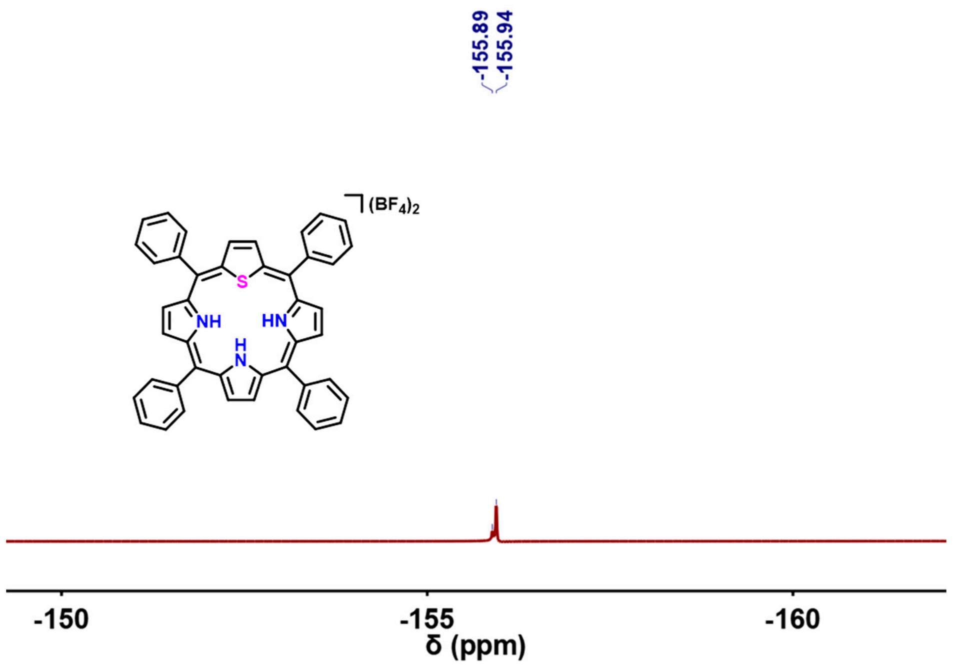

Figure S9: ^{19}F NMR spectrum (471 MHz) of **3** in $CDCl_3$ at 298 K.

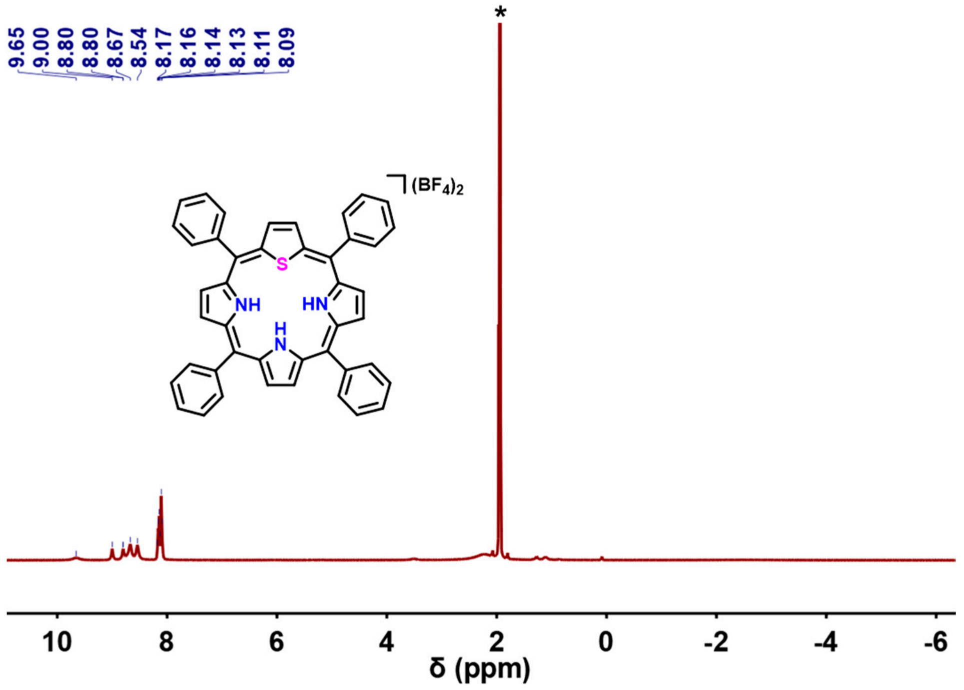

Figure S10: ^{1}H NMR spectrum (500 MHz) of **3** in CD_3CN at 298 K. Solvent signals are marked with an asterisk (*).

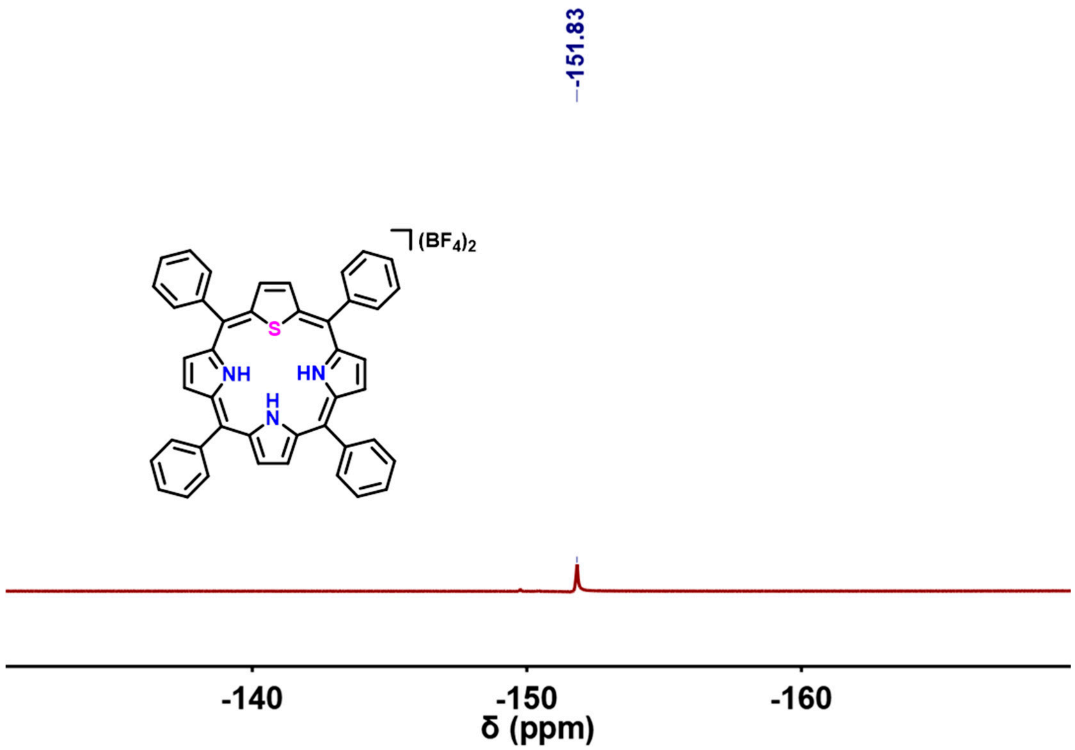

Figure S11: ^{19}F NMR spectrum (471 MHz) of **3** in CD_3CN at 298 K.

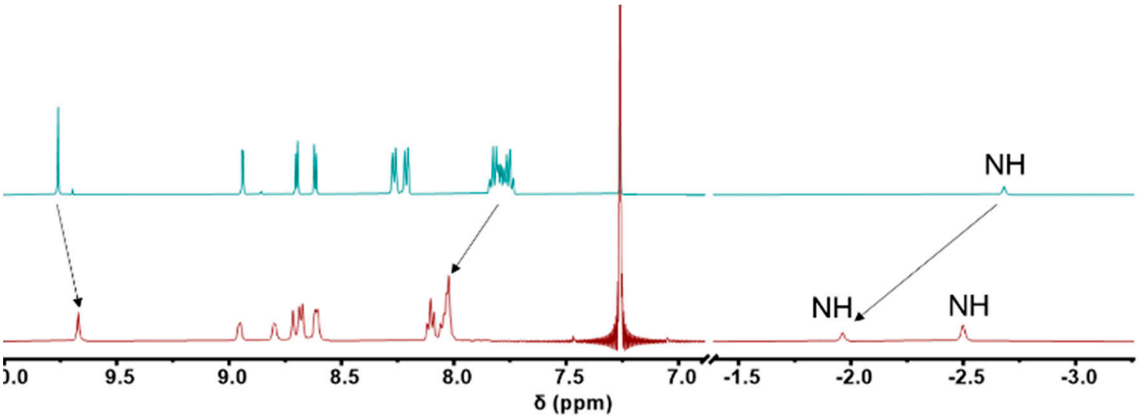

Figure S12: ^{1}H NMR spectrum (500 MHz, $CDCl_3$, 298 K) of HSTTP **1** (Top) and diacids **3** (Bottom).

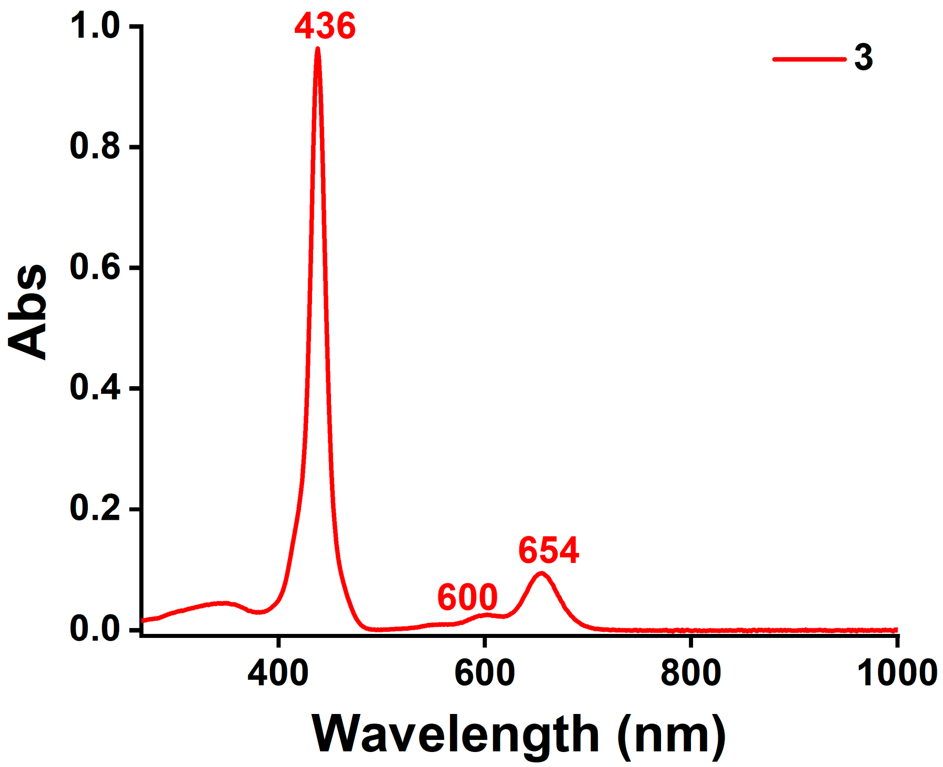

Figure S13: UV-vis absorption spectra of **3** in DCM solution.

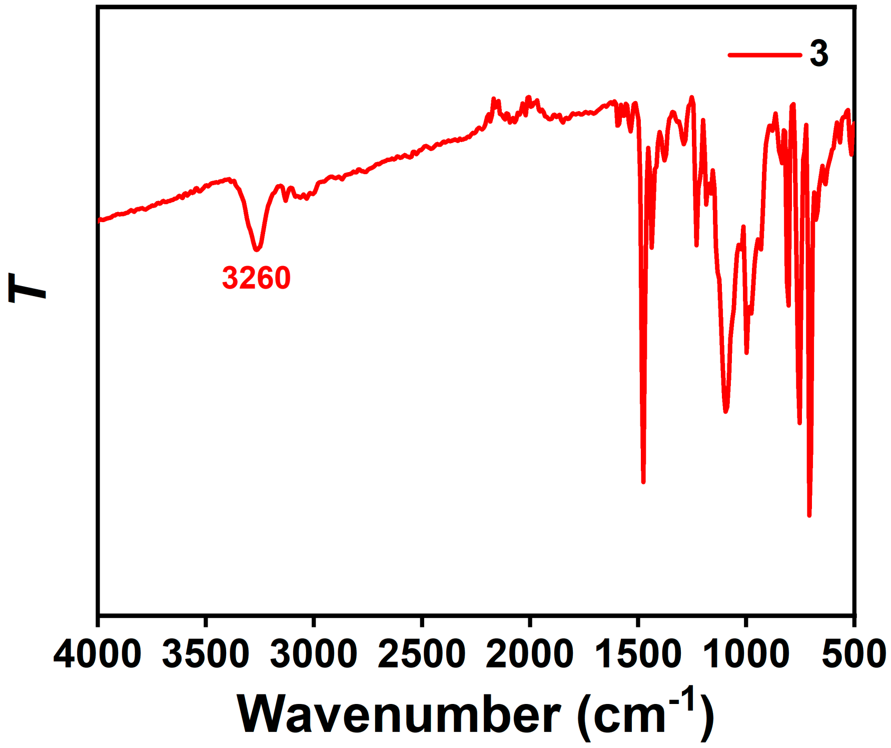

Figure S14: ATR-IR spectrum of **3** in crystalline material.

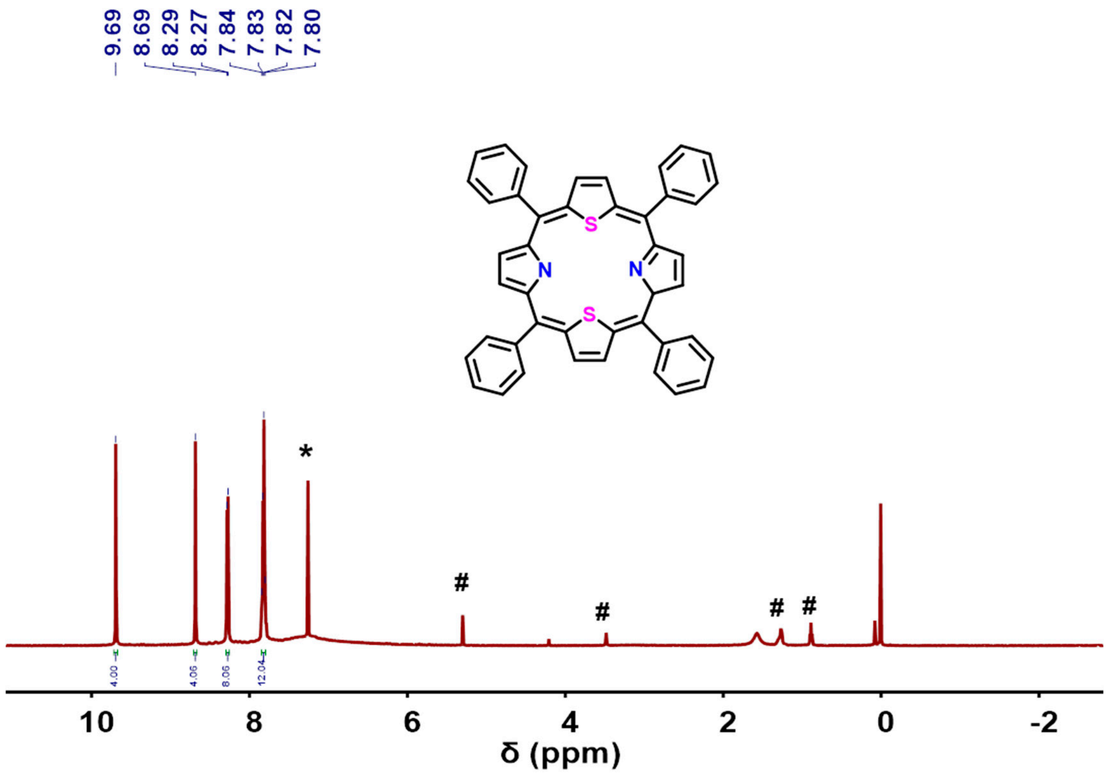

Figure S15: ^{1}H NMR spectrum (500 MHz) of **4** in $CDCl_3$ at 298 K. Solvent signals are marked with an asterisk (*). Other signals are marked with an asterisk (#).

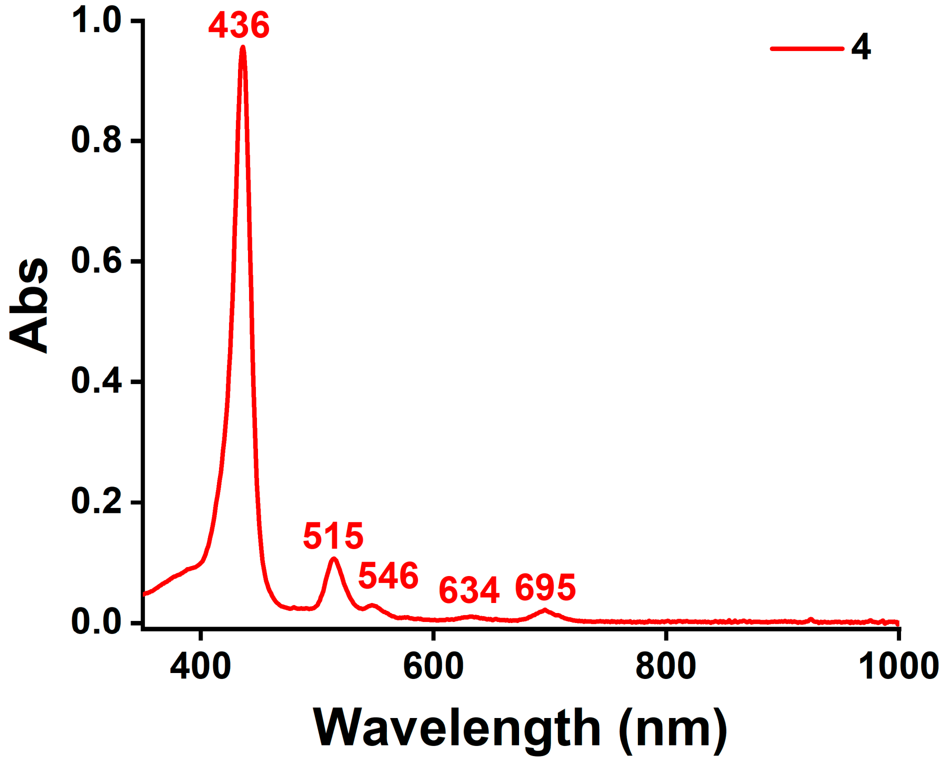

Figure S16: UV-vis absorption spectra of **4** in $CHCl_3$ solution.

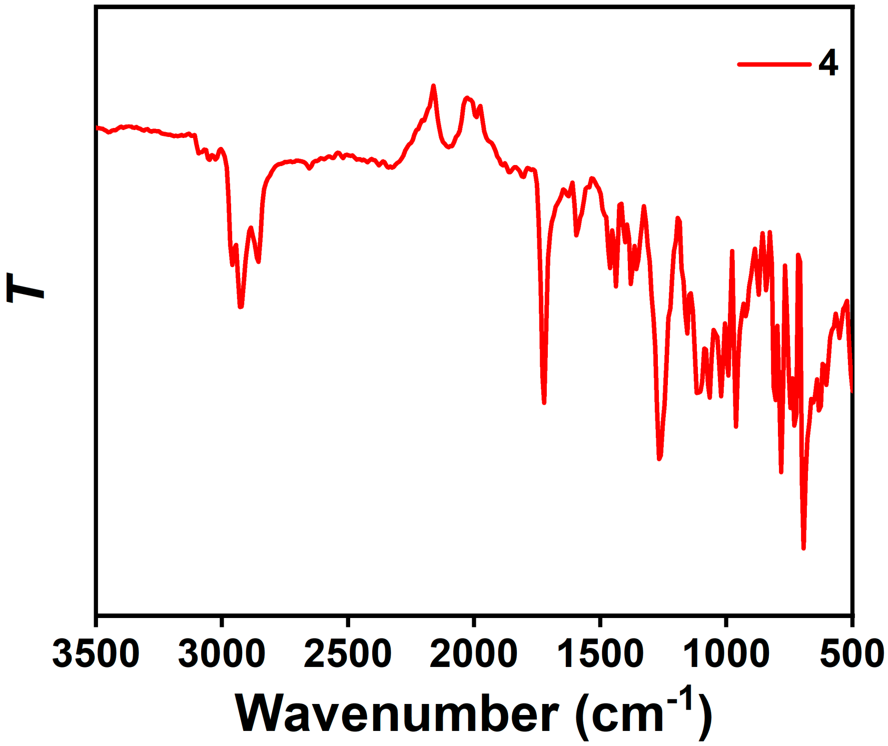

Figure S17: ATR-IR spectrum of **4** in crystalline material.

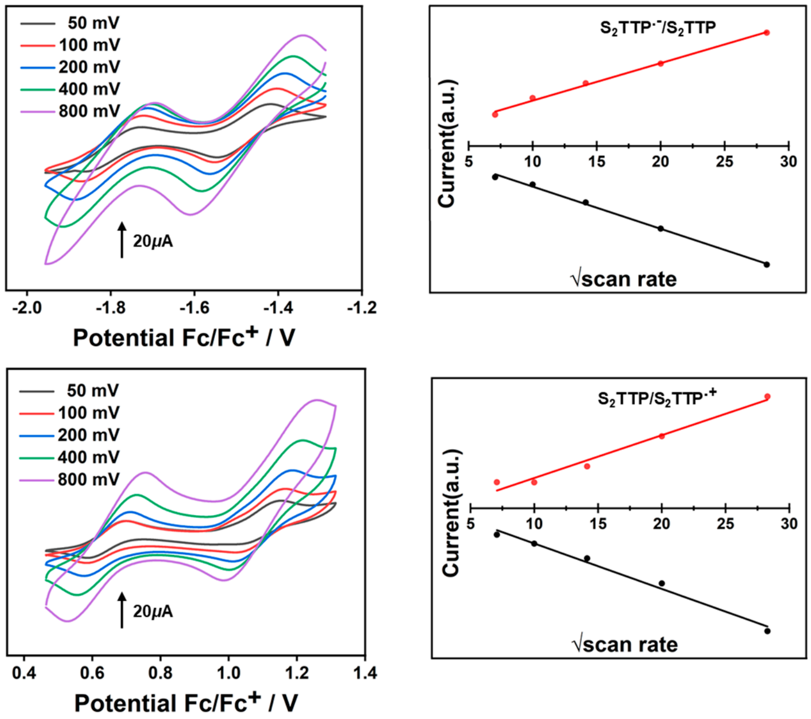

Figure S18: Left Cyclic voltammograms of **4** in DCM (0.1 M $^{n}Bu_4NPF_6$) at different scan rates. (top) S_2TTP^-/S_2TTP redox couple; (bottom) S_2TTP/S_2TTP^+ redox couple. Right: Linear dependence of the current Ifp of the forward peak on the square root of the scan rate. (top) S_2TTP^-/S_2TTP redox couple; (bottom) S_2TTP/S_2TTP^+ redox couple

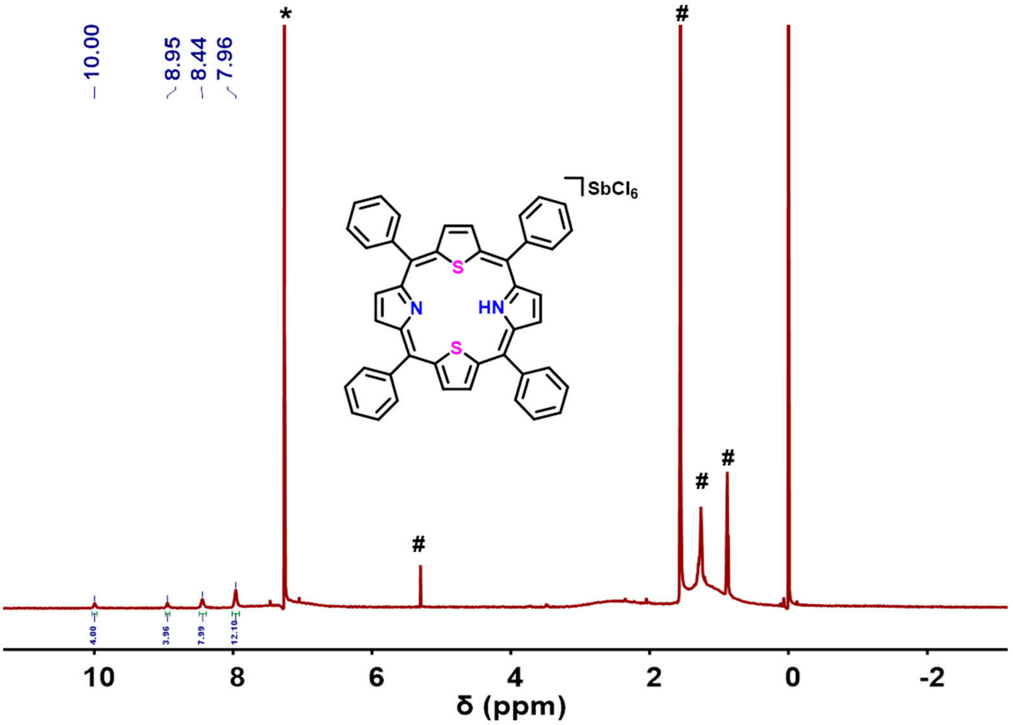

Figure S19: ^{1}H NMR spectrum (500 MHz) of **5** in $CDCl_3$ at 298 K. Solvent signals are marked with an asterisk (*). Other signals are marked with an asterisk (#).

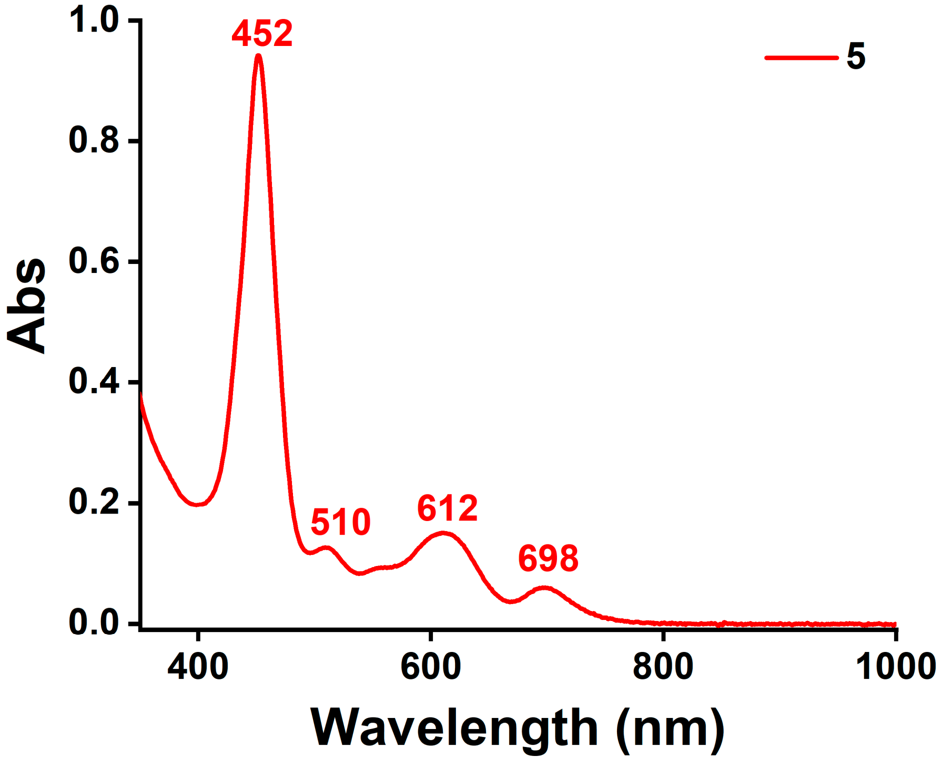

Figure S20: UV-vis absorption spectra of **5** in $CHCl_3$ solution.

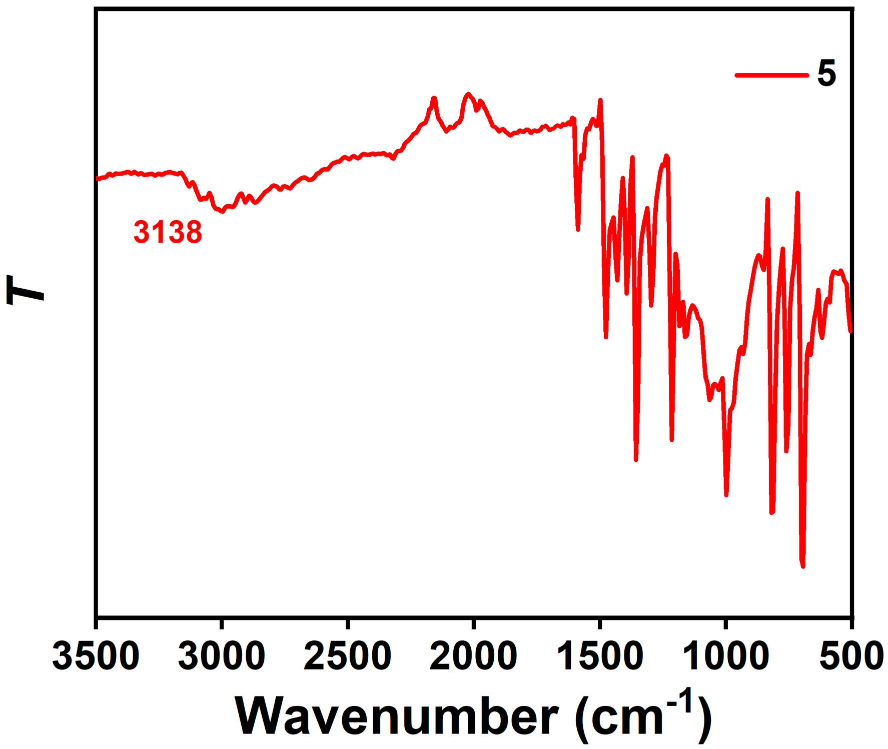

Figure S21: ATR-IR spectrum of **5** in crystalline material.

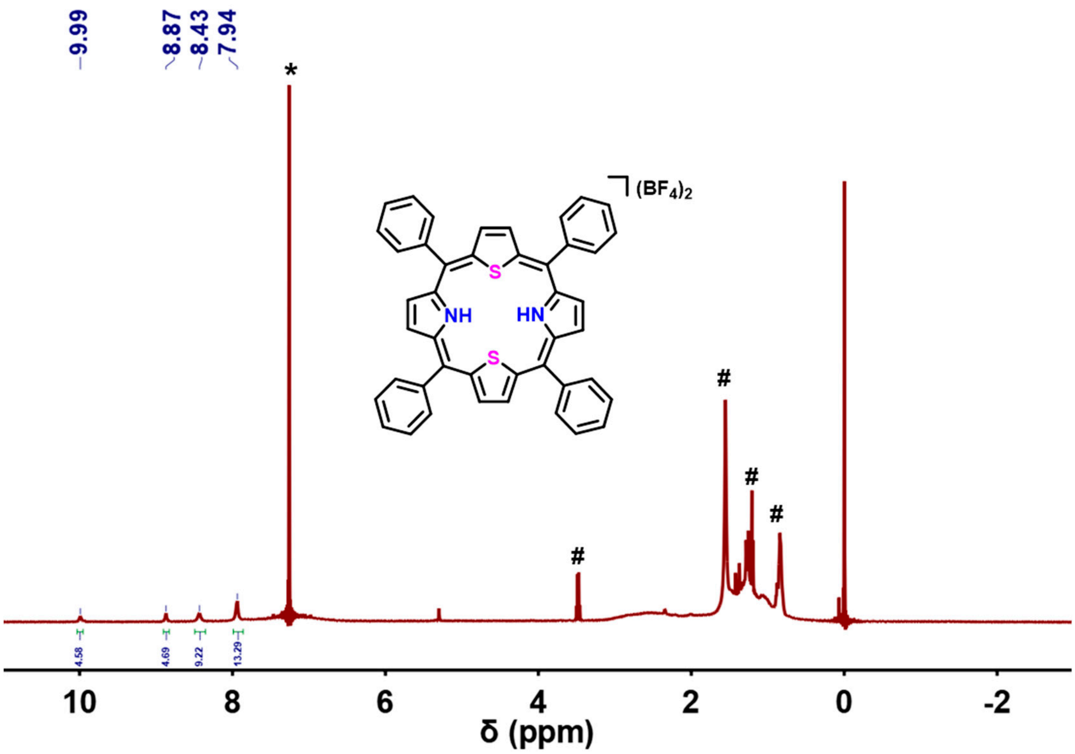

Figure S22: ^{1}H NMR spectrum (500 MHz) of **6** in $CDCl_3$ at 298 K. Solvent signals are marked with an asterisk (*). Other signals are marked with an asterisk (#).

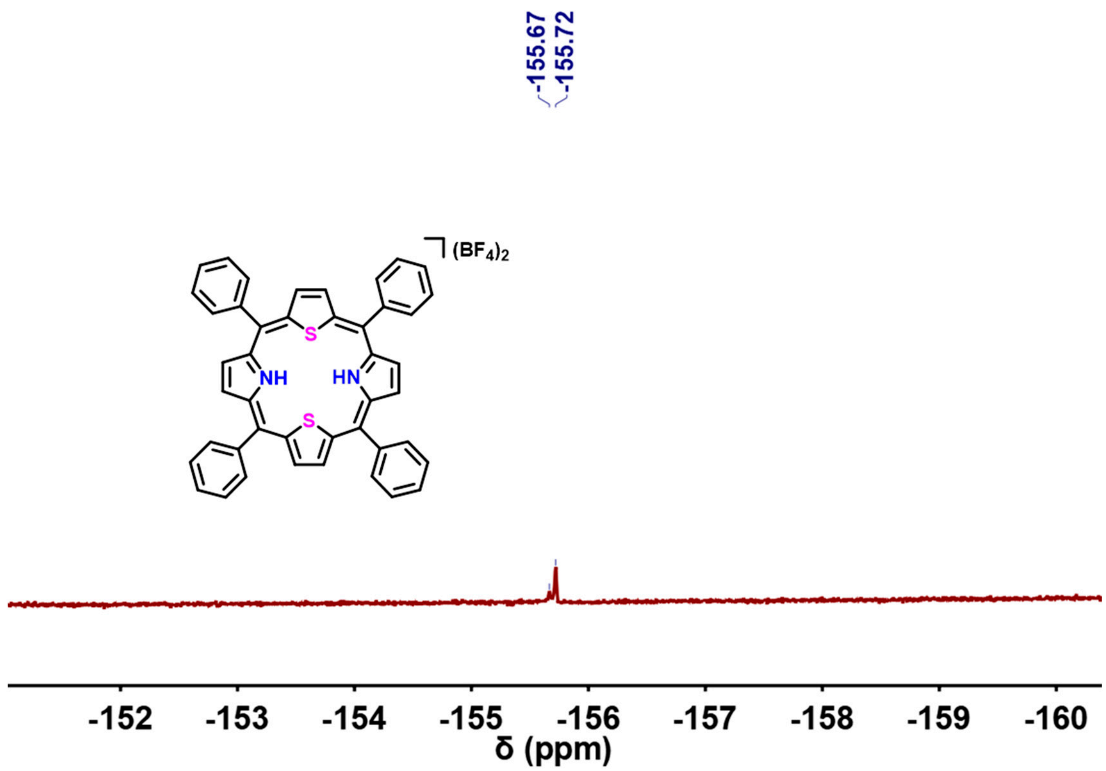

Figure S23: ^{19}F NMR spectrum (471 MHz) of **6** in $CDCl_3$ at 298 K.

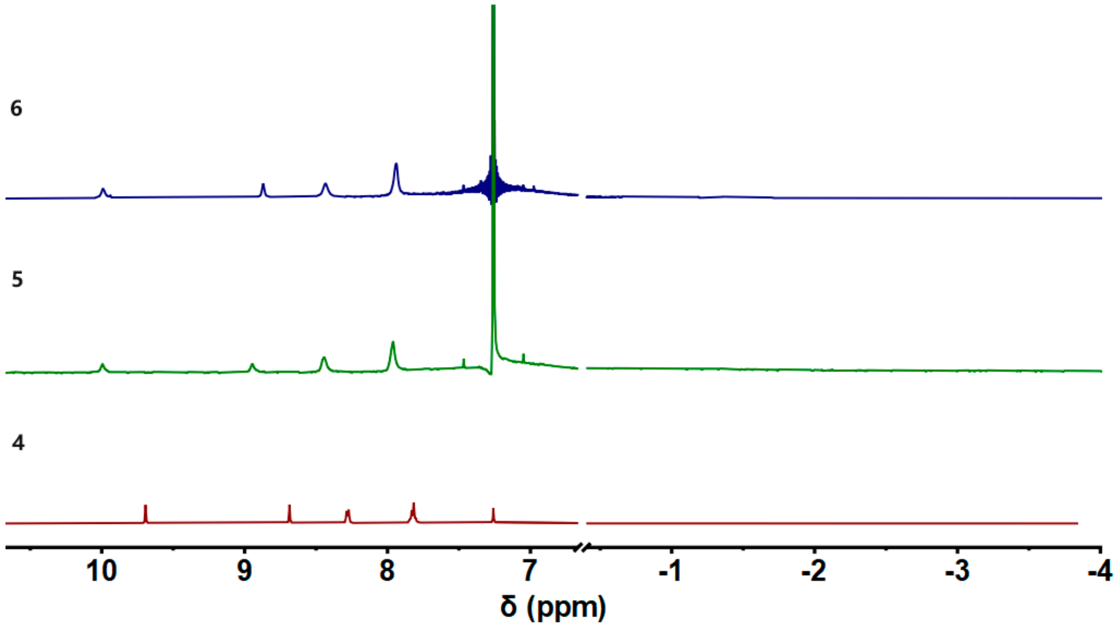

Figure S24: ^{1}H NMR spectrum (500 MHz, $CDCl_3$, 298 K) of S_2TTP **4**, monoacid **5** and diacids **6**.

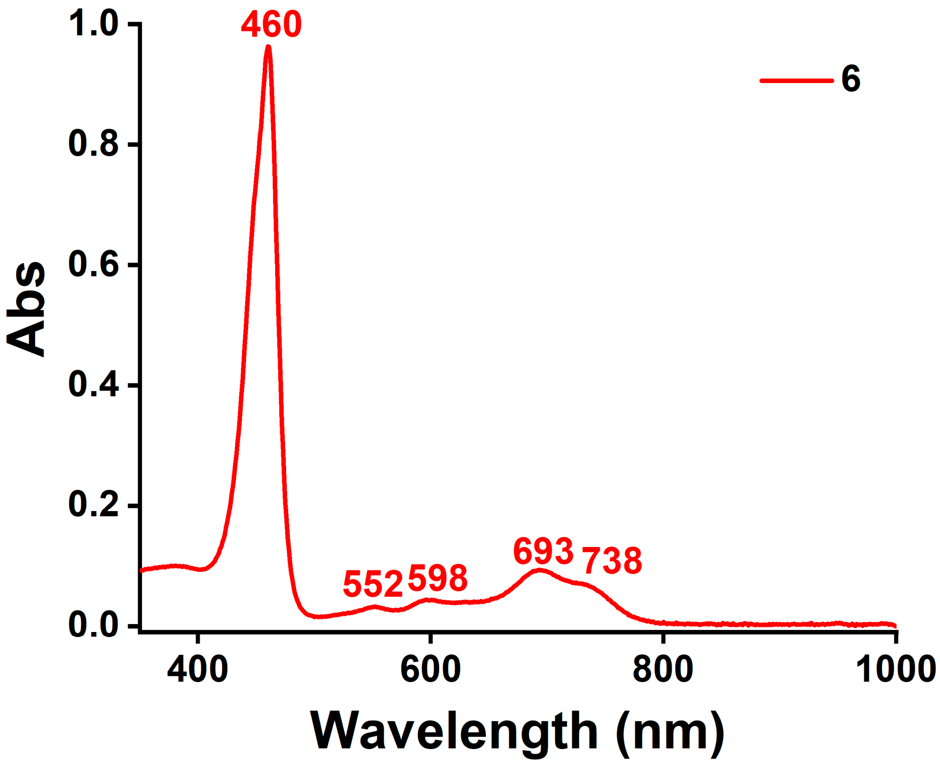

Figure S25: UV-vis absorption spectra of **6** in $CHCl_3$ solution.

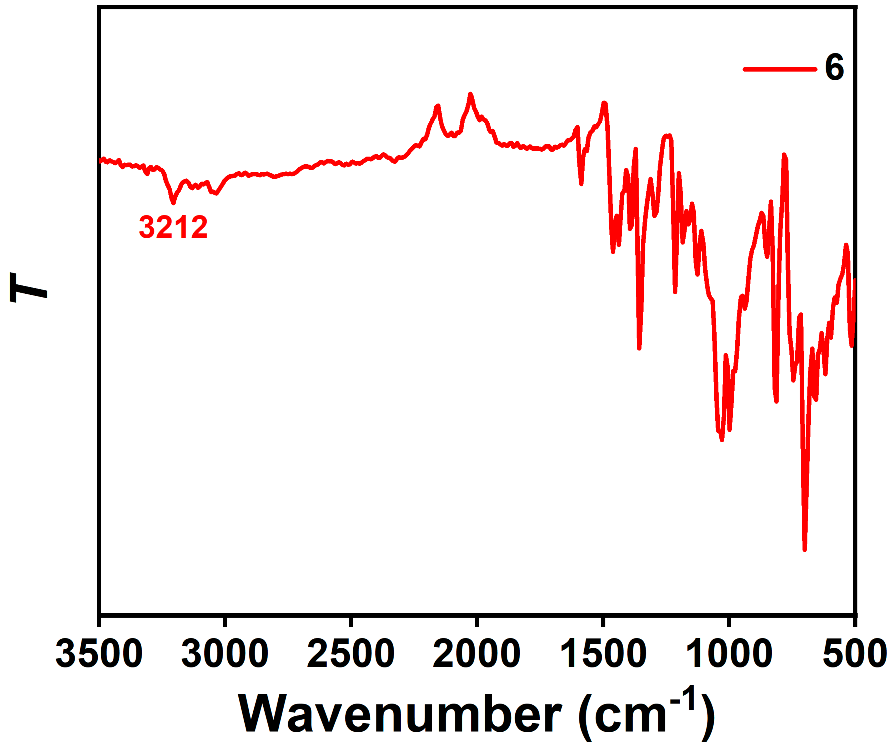

Figure S26: ATR-IR spectrum of **6** in crystalline material.

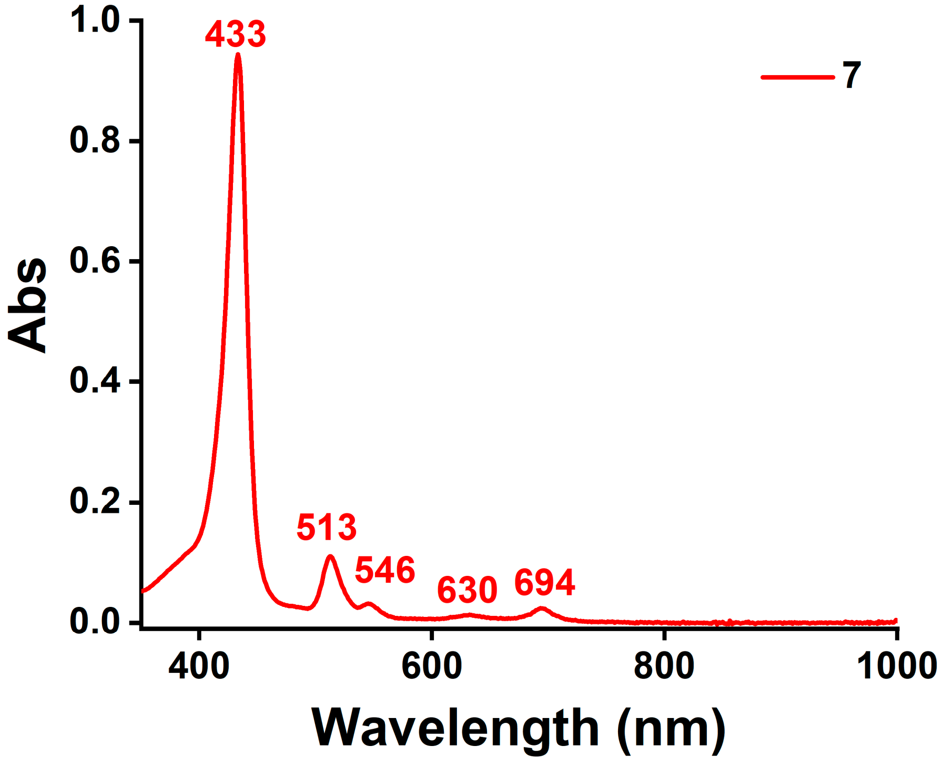

Figure S27: UV-vis absorption spectra of **7** in THF solution.

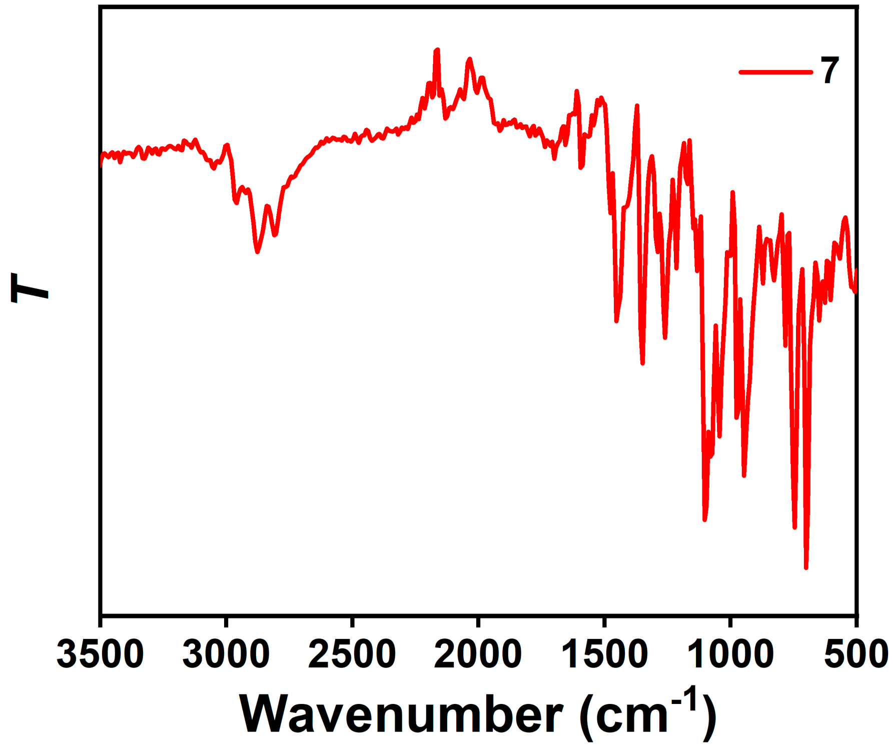

Figure S28: ATR-IR spectrum of **7** in crystalline material.

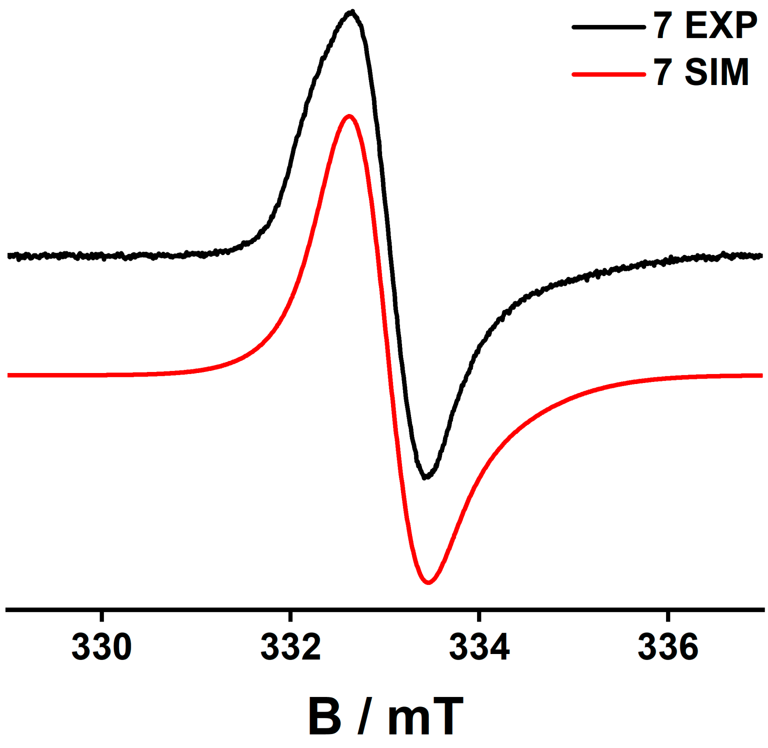

Figure S29: X-band EPR spectrum record at 9.34 GHz, microwave power 1 mW of **7** in 2-MeTHF solution at 106K. The red trace show the simulation with $g = 2.003$.

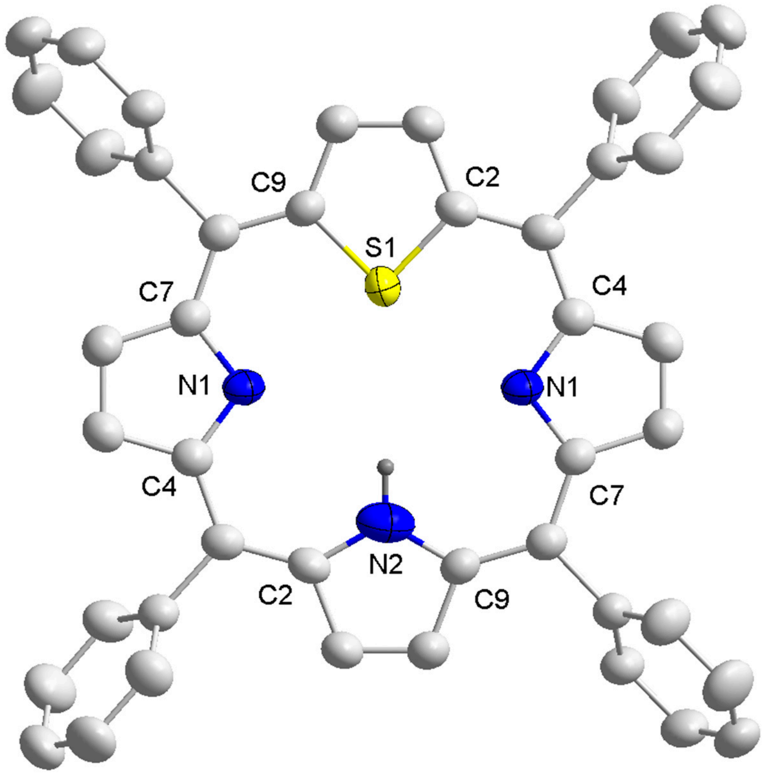

Figure S30: Plot (50% probability thermal ellipsoids) of the molecular structure of **1** (most hydrogen atoms omitted for clarity). Selected bond lengths [Å] and angles [°]: S1-C9 1.713(2), S1-C2 1.687(2), N1-C4 1.360(2), N1-C7 1.366(2), N2-C9 1.358(7), N2-C2 1.374(7), ∠C2-S1-C9 88.46(12), ∠C4-N1-C7 105.91(15), ∠C2-N2-C9 120.5(4).

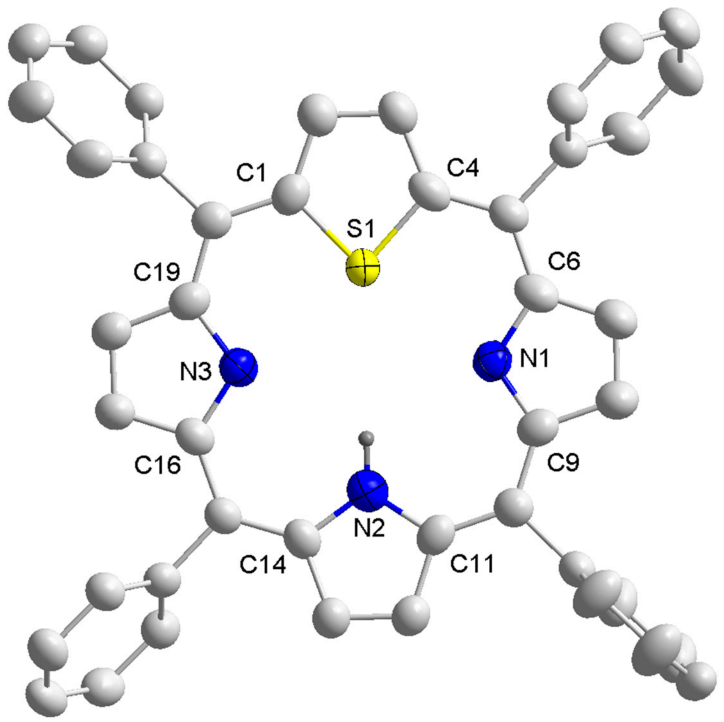

Figure S31: Plot (50% probability thermal ellipsoids) of the molecular structure of **2** (most hydrogen atoms and cation omitted for clarity). Selected bond lengths [Å] and angles [°]: S1-C1 1.729(4), S1-C4 1.721(3), N1-C6 1.374(6), N1-C9 1.369(6), N2-C11 1.384(8), N2-C14 1.374(7), N3-C16 1.392(7), N3-C19 1.392(8), ∠ C1-S1-C4 85.72(19), ∠ C6-N1-C9 111.8(4), ∠ C11-N2-C14 111.1(4), ∠ C16-N3-C19 110.7(4).

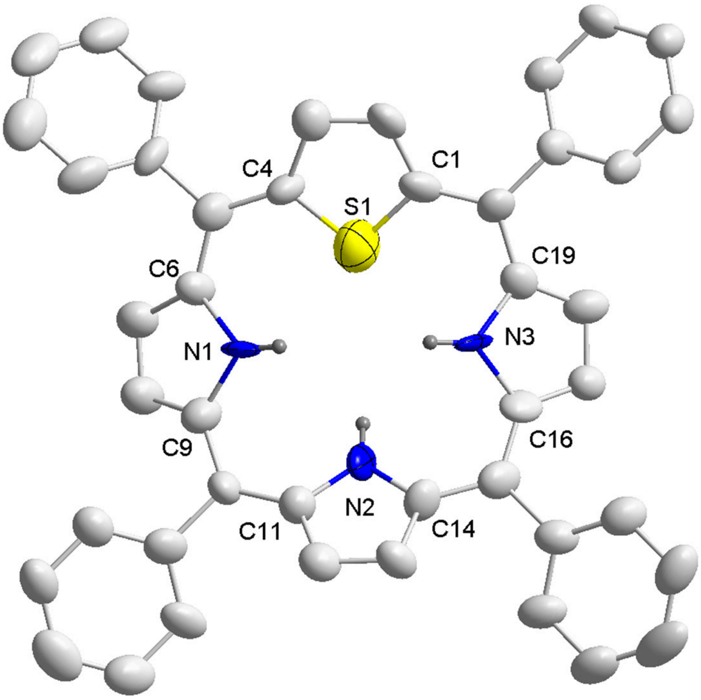

Figure S32: Plot (50% probability thermal ellipsoids) of the molecular structure of **3** (most hydrogen atoms and anion omitted for clarity). Selected bond lengths [Å] and angles [°]: S1-C1 1.676(15), S1-C4 1.686(16), N1-C6 1.519(18), N1-C9 1.489(18), N2-C11 1.455(19), N2-C14 1.448(17), N3-C16 1.468(18), N3-C19 1.472(17), ∠C1-S1-C4 93.0(8), ∠C6-N1-C9 99.2(11), ∠C11-N2-C14 102.5(12), ∠C16-N3-C19 101.0(12).

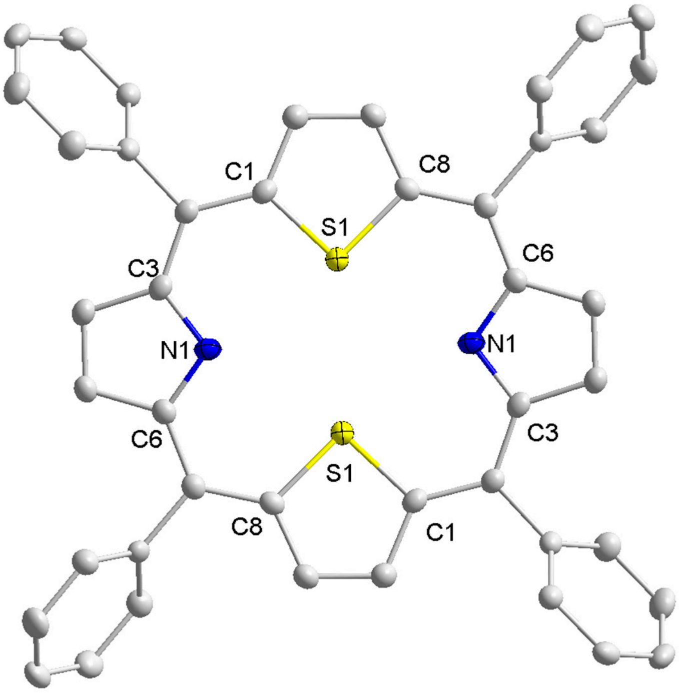

Figure S33: Plot (50% probability thermal ellipsoids) of the molecular structure of **4** (most hydrogen atoms omitted for clarity). Selected bond lengths [Å] and angles [°]: S1-C1 1.743(2), S1-C8 1.741(2), N1-C3 1.366(3), N1-C6 1.362(3), ∠C1-S1-C8 91.97(10), ∠C3-N1-C6 106.33(16).

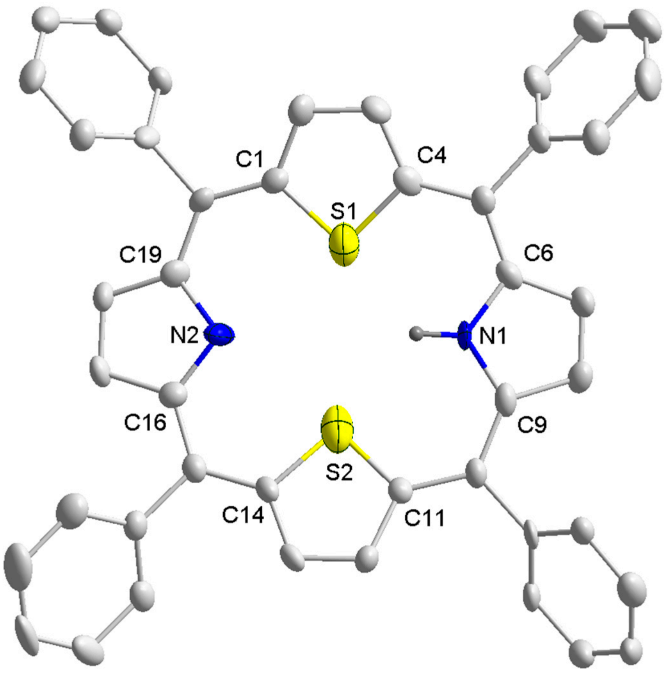

Figure S34: Plot (50% probability thermal ellipsoids) of the molecular structure of **5** (most hydrogen atoms and anion omitted for clarity). Selected bond lengths [Å] and angles [°]: S1-C1 1.689(7), S1-C4 1.686(7), S2-C11 1.675(7), S2-C14 1.663(7), N1-C6 1.364(8), N1-C9 1.376(8), N2-C16 1.368(9), N2-C19 1.384(8), ∠C1-S1-C4 92.0(4), ∠C11-S2-C14 92.6(4), ∠C6-N1-C9 109.7(6), ∠C16-N2-C19 104.7(6).

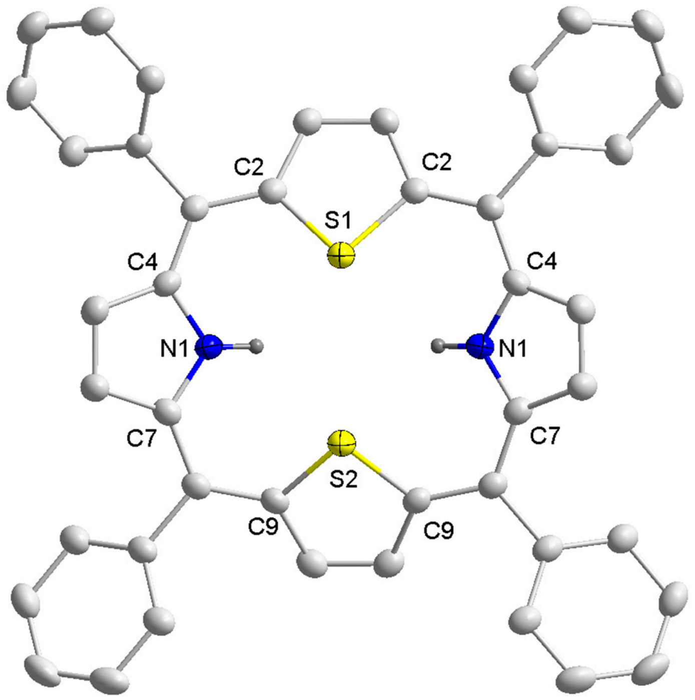

Figure S35: Plot (50% probability thermal ellipsoids) of the molecular structure of **6** (most hydrogen atoms and anion omitted for clarity). Selected bond lengths [Å] and angles [°]: S1-C2 1.743(3), S2-C9 1.737(3), N1-C4 1.374(4), N1-C7 1.377(4), ∠C2-S1-C2 92.6(2), ∠C9-S2-C9 93.0(2), ∠C4-N1-C7 110.4(3).

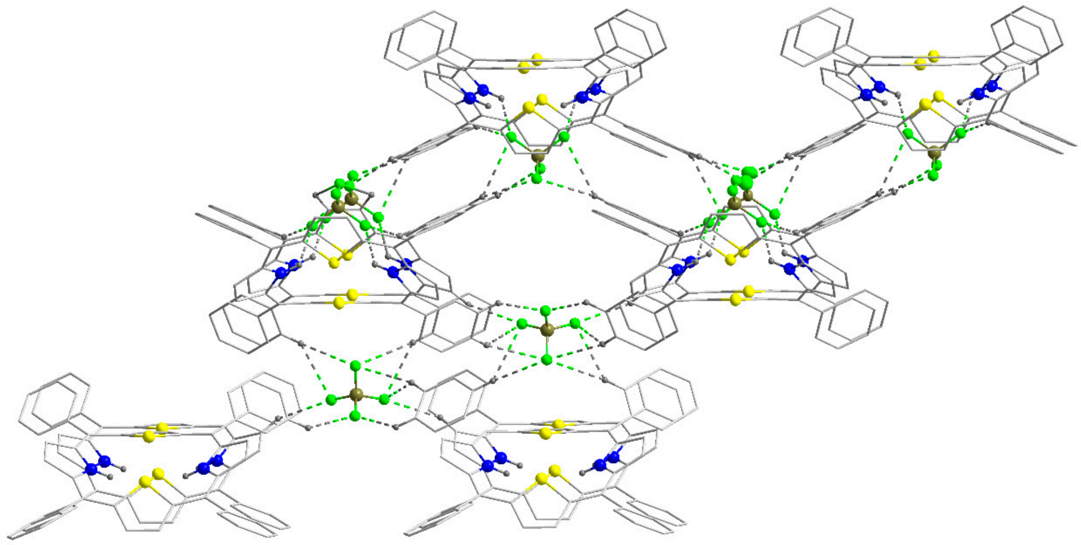

Figure S36: 3D arrangement in the crystal lattice showing interactions with the $C_{(Ph)}$-H···BF_4 anion. Solvent molecules and hydrogen atoms are omitted for clarity, except for the NH.

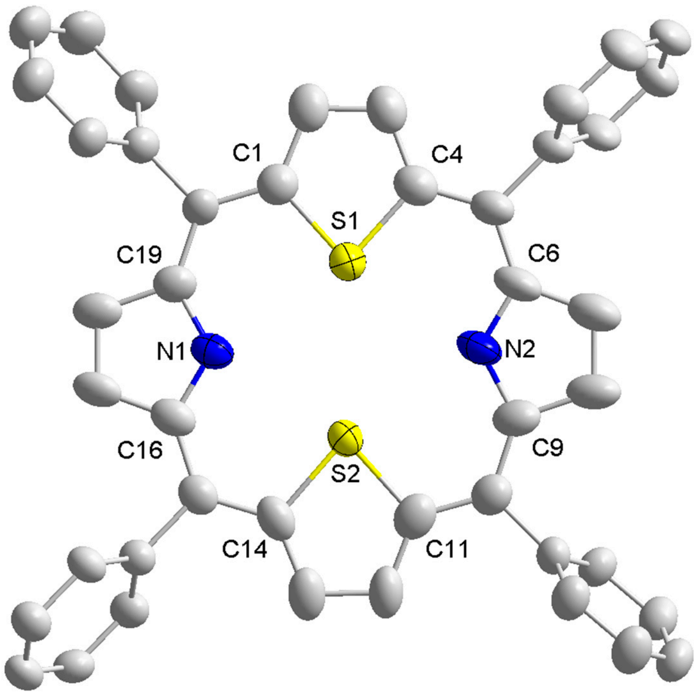

Figure S37: Plot (50% probability thermal ellipsoids) of the molecular structure of **7** (most hydrogen atoms and cation omitted for clarity). Selected bond lengths [Å] and angles [°]: S1-C1 1.805(12), S1-C4 1.769(12), S2-C11 1.808(11), S2-C14 1.837(11), N1-C16 1.340(2), N1-C19 1.330(2), N2-C6 1.294(19), N2-C9 1.370(2), ∠C1-S1-C4 84.1(5), ∠C11-S2-C14 82.6(5), ∠C16-N1-C19 119.6(13), ∠C6-N2-C9 121.2(14).

Table S1. Crystal data and refinement details for compounds **1**, **2**, **3**, **4**, **5**, **6** and **7**.

Compound	1	2	3
	HSTTP	[Na(Cryptand)](HSTTP)	$(H_3STTP)(BF_4)_2$
moiety formula	$C_{44}H_{29}N_3S$	$C_{18}H_{36}N_2NaO_6 \cdot C_{44}H_{29}N_3S \cdot 0.8(C_2H_3N)$	$C_{44}H_{31}N_3S \cdot C_2H_3N \cdot 2(BF_4)$
empirical formula	$C_{44}H_{29}N_3S$	$C_{63.6}H_{67.4}N_{5.8}NaO_6S$	$C_{46}H_{34}B_2F_8N_4S$
T (K)	301	150	150
formula weight	315.88	1064.08	848.45
crystal size [mm³]	0.23×0.17×0.13	0.15×0.13×0.12	0.17×0.16×0.11
crystal system	Monoclinic	Monoclinic	Monoclinic
space group	*P*2/c	*P*2/n	$P2_1$
a [Å]	12.089(4)	11.6796(4)	9.464(3)
b [Å]	11.714(4)	20.8382(9)	21.762(8)
c [Å]	11.761(4)	23.3113(8)	10.031(4)
α [°]	90	90	90
β [°]	101.744(12)	101.3290(10)	107.477(11)
γ [°]	90	90	90
V [Å³]	1630.6(10)	5563.0(4)	1970.6(12)
Z	4	4	2
ρ [g·cm⁻³]	1.287	1.270	1.430
F(000)	660	2258	872
μ [mm⁻¹]	0.137	0.124	0.161
T_{min} / T_{max}	0.6538/0.7457	0.0248/ 0.0479	0.0427 / 0.0964
θ-range [°]	2.446-25.749	1.955-25.750	2.129 - 25.000
hkl-range	±14, -13 -14, ±14	±14, -25 -23, -28-27	±11, ±25, ±11
measured refl.	15601	46281	13531
unique refl. [R_{int}]	3123 [0.0451]	10643 [0.0576]	6622 [0.1686]
observed refl. ($I > 2(I)$)	2263	7558	2690
data / restr / param.	3123/1/230	10643/942/983	6622/235/548
goodness-of-fit (F^2)	1.055	1.056	1.010
R_1, wR_2 ($I > 2(I)$)	0.0425/0.0926	0.0689/0.1670	0.1006/0.2209
R_1, wR_2 (all data)	0.0677/0.1085	0.0980/0.1876	0.2298/0.3073
res. el. dens. [e·Å⁻³]	-0.302/0.516	-0.464/0.684	-0.510/0.491

**Compound**	**4** S_2TTP	**5** $(HS_2TTP)SbCl_6$	**6** $(H_2S_2TTP)(BF_4)_2$
moiety formula	$C_{44}H_{28}N_2S_2$	$C_{44}H_{29}N_2S_2\cdot SbCl_6\cdot C_4H_8O\cdot CH_3CN$	$C_{22}H_{15}NS\cdot 2(B_{0.5}F_2)$
empirical formula	$C_{44}H_{28}N_2S_2$	$C_{50}H_{40}Cl_6N_3OS_2Sb$	$C_{22}H_{15}BF_4NS$
T (K)	150	150	150
formula weight	648.80	1097.42	412.22
crystal size [mm³]	0.28×0.2×0.18	0.2×0.18×0.15	0.12×0.12×0.1
crystal system	Monoclinic	Triclinic	Orthorhombic
space group	*P*2/c	*P*-1	*Pnma*
a [Å]	11.9829(5)	12.9291(8)	32.1452(15)
b [Å]	11.5801(4)	13.9689(10)	16.9625(7)
c [Å]	11.7140(6)	14.0358(9)	8.2039(3)
α [°]	90	103.380(5)	90
β [°]	102.426(2)	94.423(5)	90
γ [°]	90	100.957(5)	90
V [Å³]	1587.39(12)	2401.6(3)	4473.3(3)
Z	2	2	8
ρ [g·cm⁻³]	1.357	1.518	1.224
F(000)	676	1108	1688
μ [mm⁻¹]	0.205	1.036	0.184
T_{min} / T_{max}	0.6871 / 0.7457	0.8824/ 1.0000	0.7030 / 0.7457
θ-range [°]	2.474 - 25.743	3.230 - 25.750	2.401 - 25.749
hkl-range	±14, -14 - 13, ±14	±15, -16 - 17, -16 - 17	-34 - 39, -19 - 20, -9 - 10
measured refl.	20693	18452	34773
unique refl. [R_{int}]	3031 [0.0607]	9159 [0.0557]	4424 [0.0847]
observed refl. ($I > 2(I)$)	2476	5507	3201
data / restr / param.	3031/0/217	9154/18/569	4424/6/274
goodness-of-fit (F^2)	1.054	1.030	1.105
R_1, wR_2 ($I > 2(I)$)	0.0400/0.0873	0.0766/0.1592	0.0626/0.1746
R_1, wR_2 (all data)	0.0548/0.0973	0.1357/0.1852	0.0867/0.1946
res. el. dens. [e·Å⁻³]	-0.342/0.669	-0.982/1.139	-0.546/0.617

**Compound**	**7**<br>[K(Cryptand)]S_2TTP
moiety formula	$C_{44}H_{28}N_2S_2 \cdot C_{18}H_{36}KN_2O_6 \cdot 0.5[C_4H_8O] \cdot 0.5[C_4H_8O]$
empirical formula	$C_{62}H_{64}KN_4O_6S_2$
T (K)	150
formula weight	1064.39
crystal size [mm³]	0.14×0.12×0.1
crystal system	Triclinic
space group	*P*-1
a [Å]	12.951(10)
b [Å]	17.598(13)
c [Å]	26.63(2)
α [°]	88.81(2)
β [°]	76.54(3)
γ [°]	86.89(3)
V [Å³]	5893(8)
Z	4
ρ [g·cm⁻³]	1.200
F(000)	2252
μ [mm⁻¹]	0.213
T_{min} / T_{max}	0.6296/0.7441
θ-range [°]	1.942 - 17.353
hkl-range	±10, ±14, ±22
measured refl.	47351
unique refl. [R_{int}]	7244 [0.1110]
observed refl. ($I > 2(I)$)	5127
data / restr / param.	7244/2728/1896
goodness-of-fit (F^2)	1.054
R_1, wR_2 ($I > 2(I)$)	0.0520/0.1162
R_1, wR_2 (all data)	0.0827/0.1346
res. el. dens. [e·Å⁻³]	-0.278/0.290

The ZINDO/S method was used to calculate the excitation energies and absorption spectra for the x-ray geometries of the compound **1-7**. RHF for **1**, **3**, **4-6** and UHF for **2** and **7** DFT calculations, ZINDO/S semiempirical method, TightSCF instruction, DIIS approximation using the auxiliary TightSCF basis set. (! RHF ZINDO/S TightSCF DIIS NoRICO).

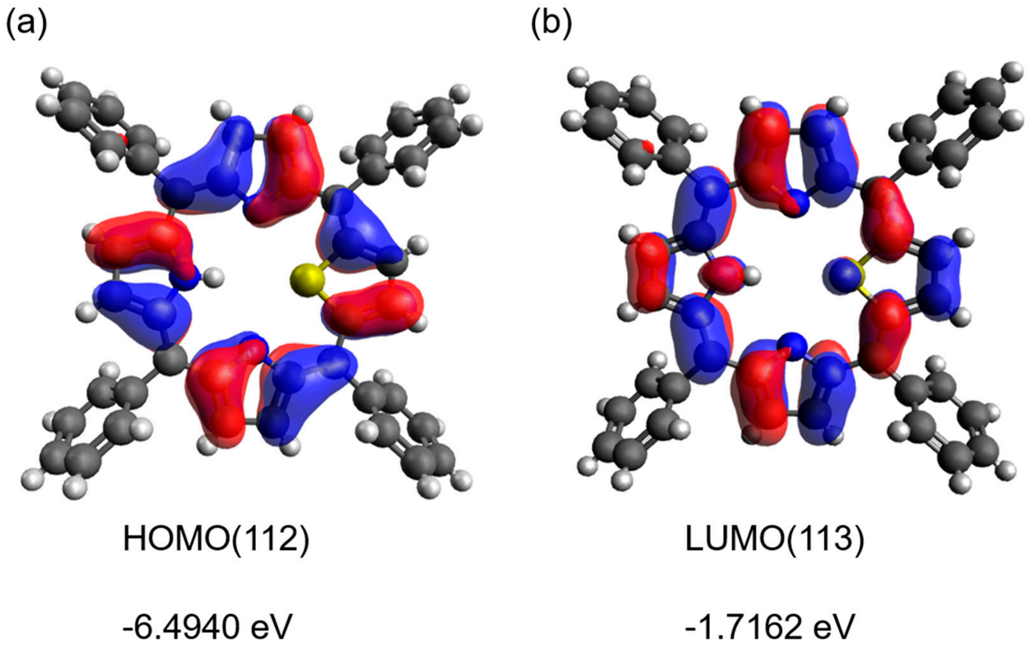

Figure S38: HOMO (a) and LUMO (b) plot for the compound **1**.

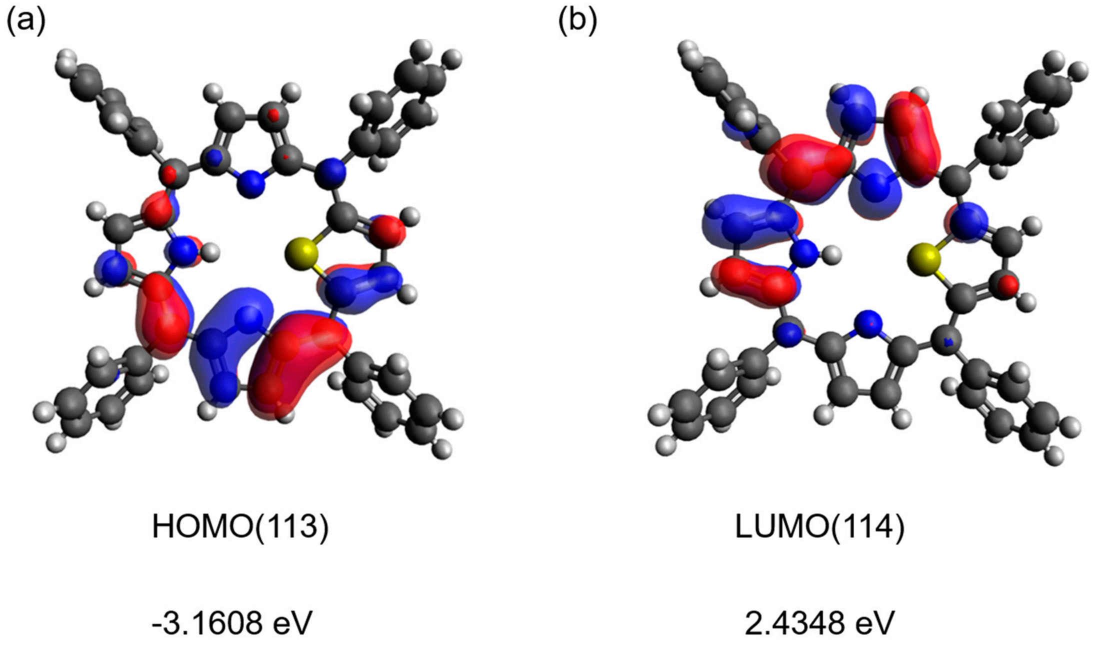

Figure S39: HOMO (a) and LUMO (b) plot for the compound **2**.

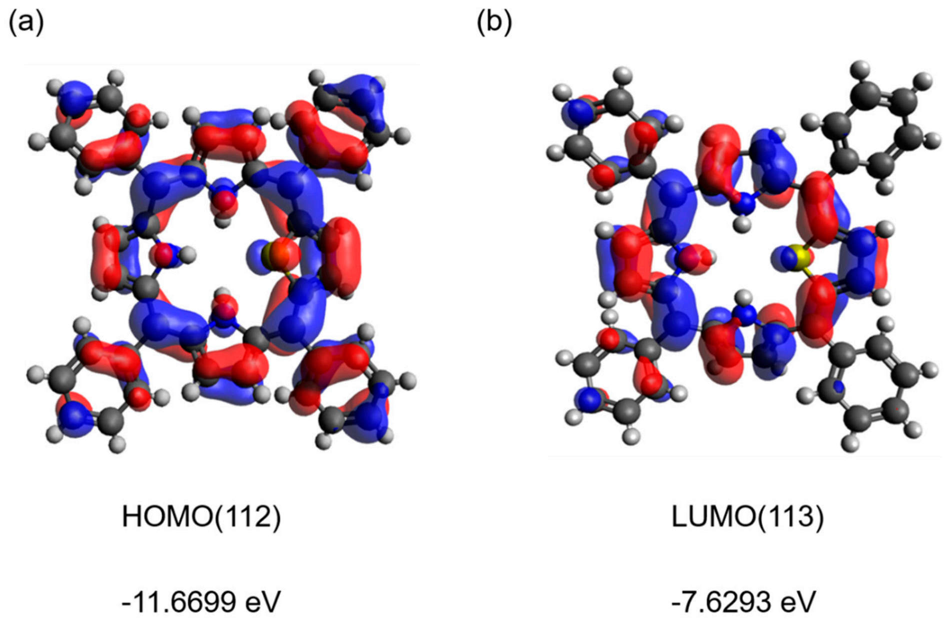

Figure S40: HOMO (a) and LUMO (b) plot for the compound **3**.

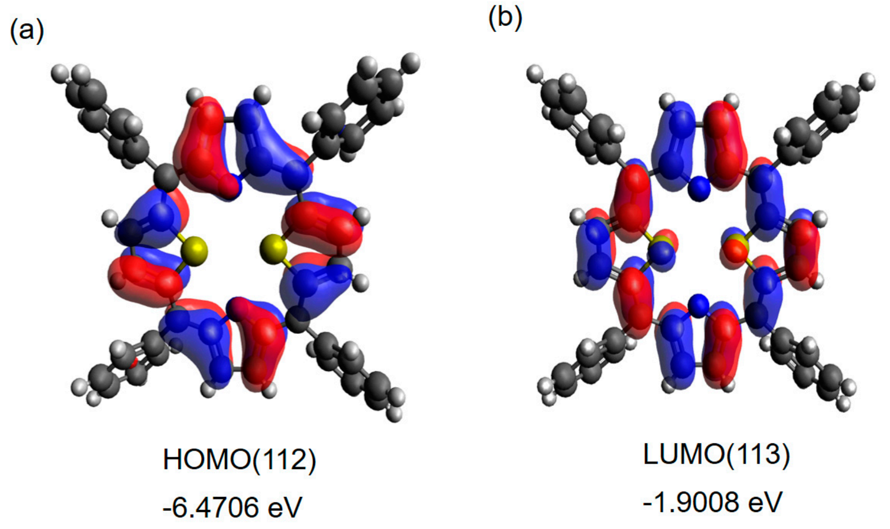

Figure S41: HOMO (a) and LUMO (b) plot for the compound **4**.

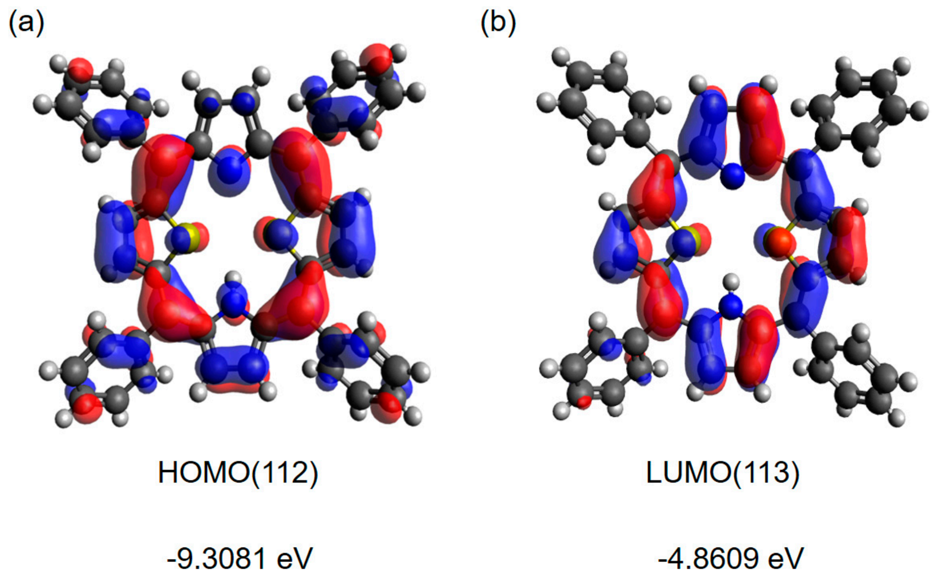

Figure S42: HOMO (a) and LUMO (b) plot for the compound **5**.

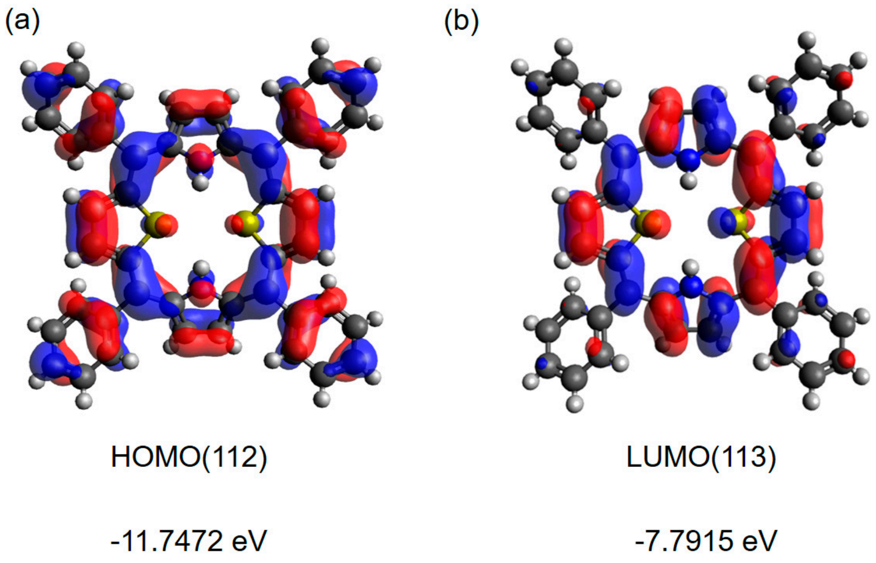

Figure S43: HOMO (a) and LUMO (b) plot for the compound **6**.

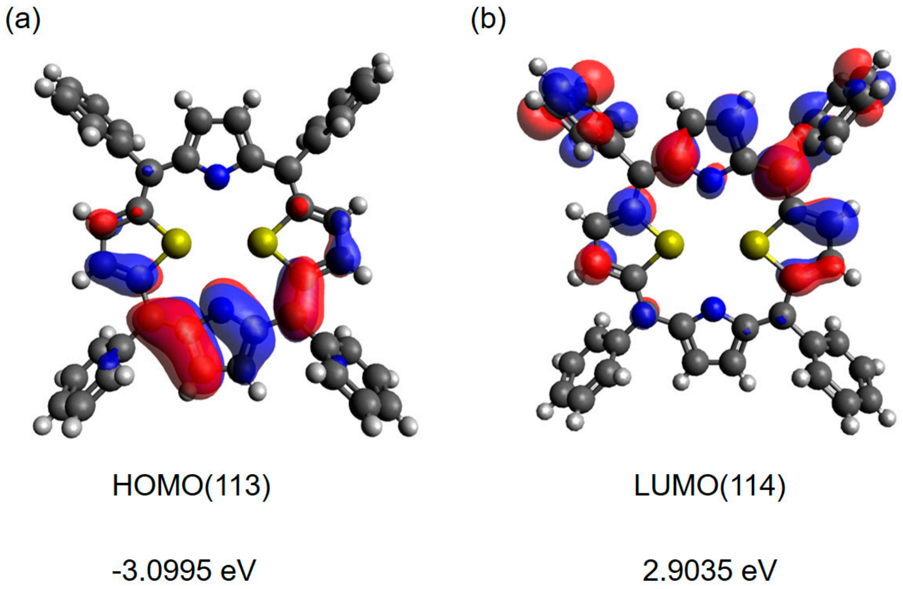

Figure S44: HOMO (a) and LUMO (b) plot for the compound **7**.

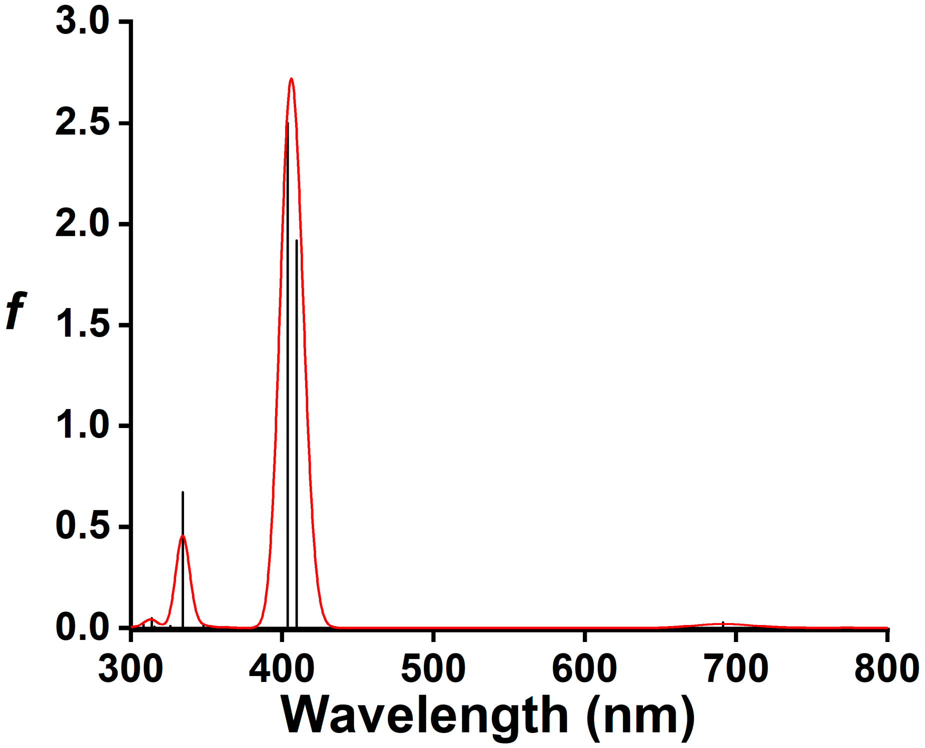

Figure S45: Calculated absorption spectra of the compound **1** obtained using the ZINDO/S method.

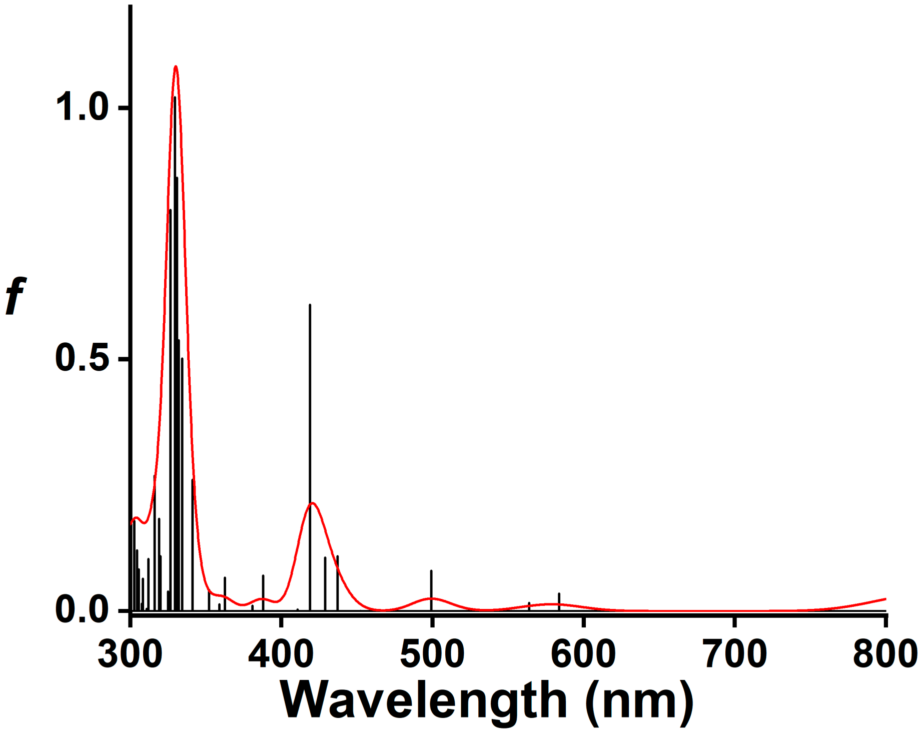

Figure S46: Calculated absorption spectra of the compound **2** obtained using the ZINDO/S method.

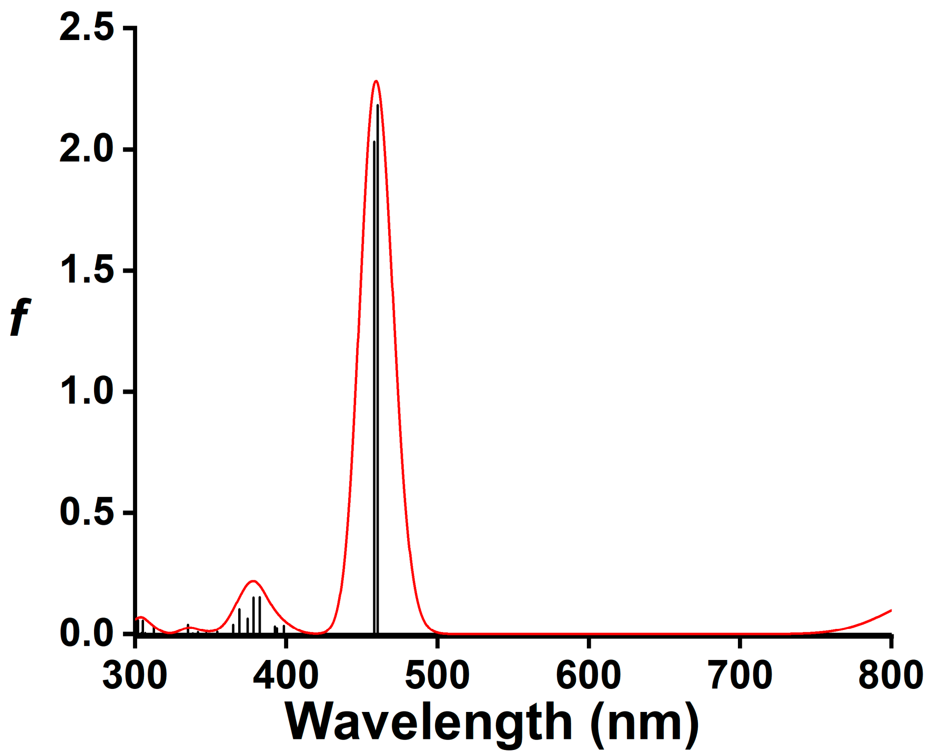

Figure S47: Calculated absorption spectra of the compound **3** obtained using the ZINDO/S method.

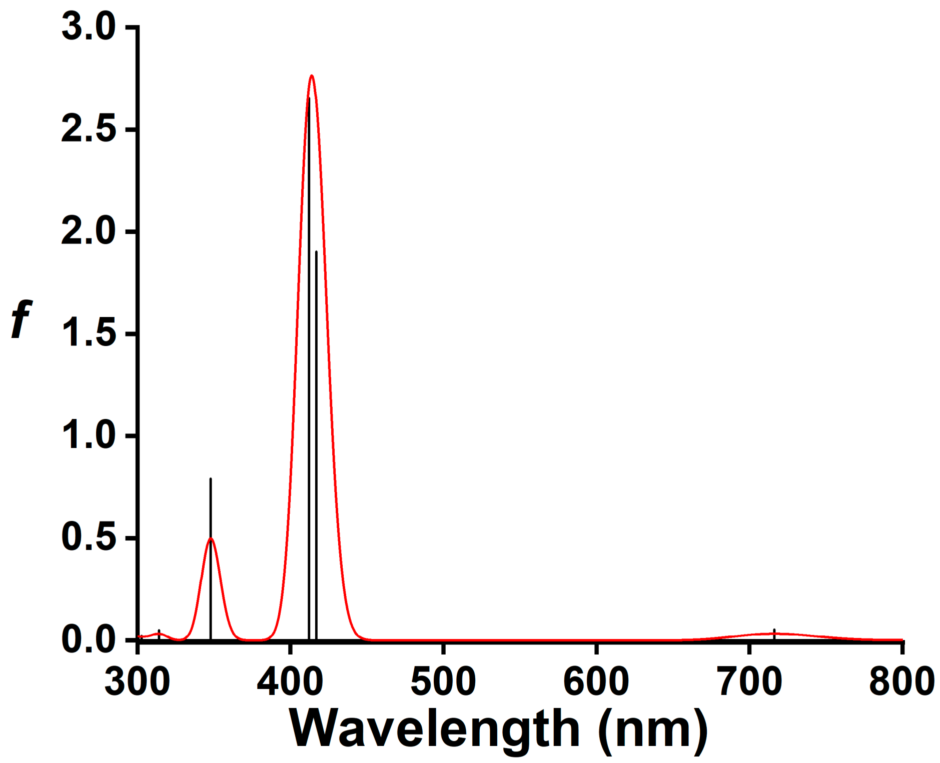

Figure S48: Calculated absorption spectra of the compound **4** obtained using the ZINDO/S method.

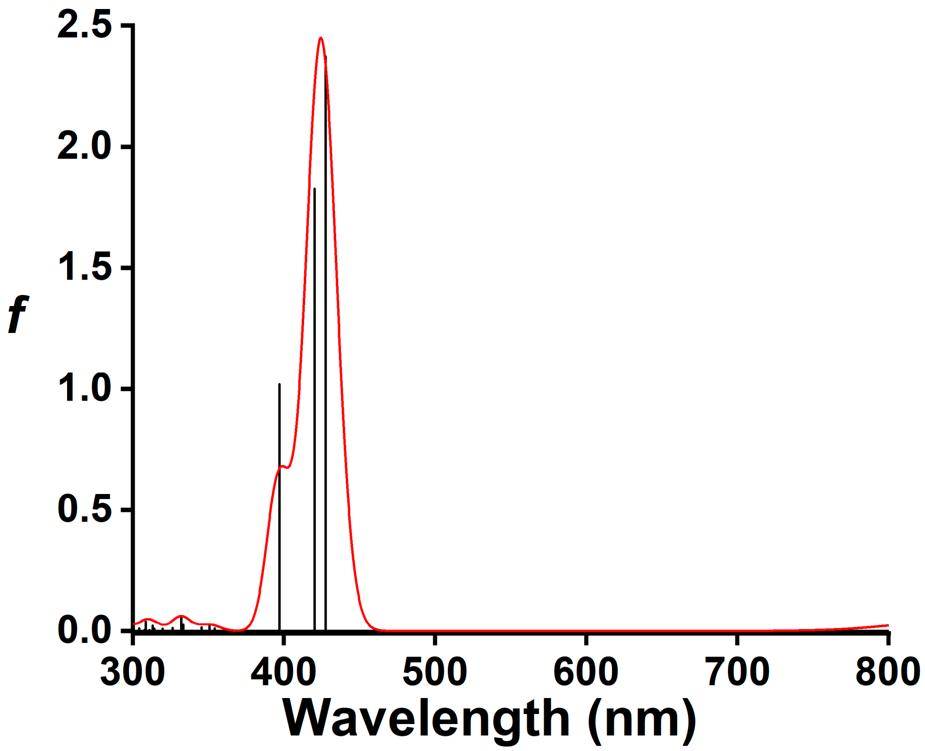

Figure S49: Calculated absorption spectra of the compound **5** obtained using the ZINDO/S method.

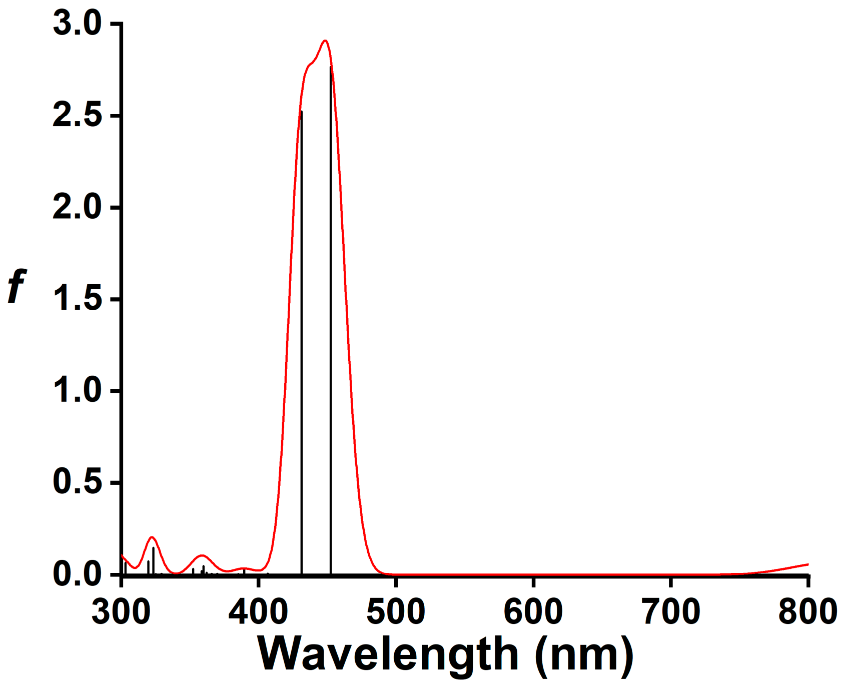

Figure S50: Calculated absorption spectra of the compound **6** obtained using the ZINDO/S method.

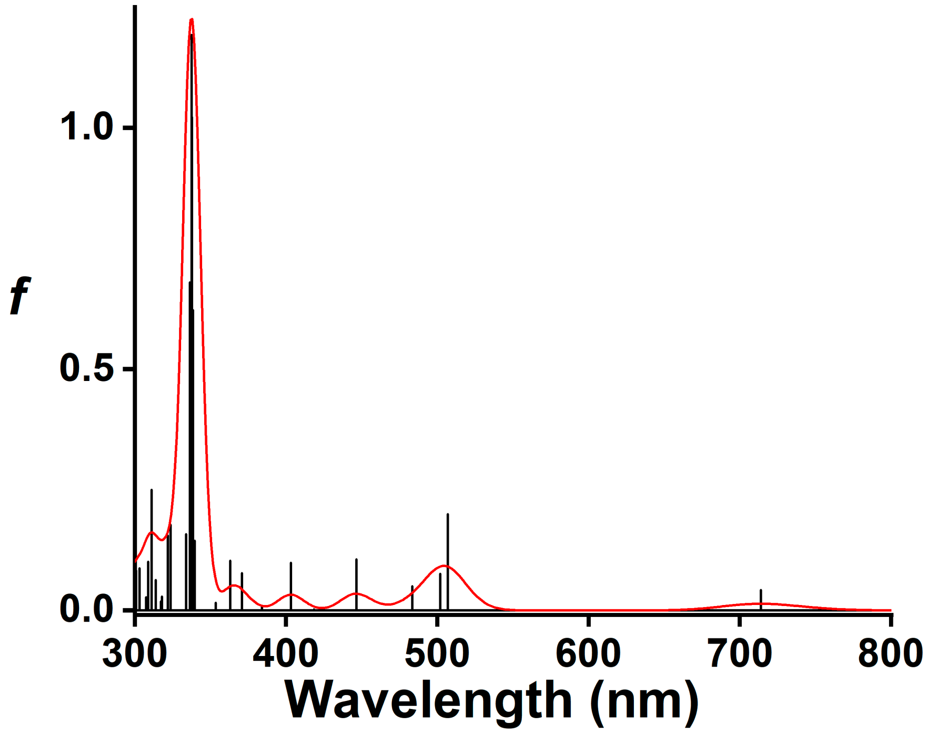

Figure S51: Calculated absorption spectra of the compound **7** obtained using the ZINDO/S method.

Table S2: Selected Results of ZINDO/S Calculations for Low-Energy *π*-*π** Transitions of compounds **1-7**.

compound	λ (nm)	*f*	Composition (weight %)[a]
**1**	691	0.03	H-1⟶L+1 (33)
			H⟶L (53)
	409	1.92	H-1⟶L (30)
			H ⟶L+1 (40)
	403	2.50	H-1 ⟶L+1 (48)
			H⟶L (30)
**2**	584	0.034	S-8⟶S (25)
			S-2⟶S (15)
	564	0.016	S-8⟶S (15)
			S-2⟶S (18)
	499	0.080	S-1⟶L (20)
			S⟶L (29)
	437	0.11	S⟶L (12)
			S-1⟶L (13)
			S-1 ⟶ L+5 (12)
	429	0.11	S-5⟶S (12)
	419	0.61	S⟶L+5 (18)
			S-5 ⟶H (10)
**3**	460	2.18	H-1⟶L (45)
			H⟶L+1 (26)
	458	2.03	H-1⟶L+1 (51)
			H⟶L (14)
**4**	716	0.054	H-1⟶L+1 (30)
			H⟶L (57)
	416	1.90	H-1⟶L (31)
			H⟶L+1 (44)
	412	2.66	H-1⟶L+1 (56)
			H⟶L (29)
**5**	427	2.37	H-1⟶L (39)
			H⟶L+1 (45)
	420	1.83	H-1⟶L+1 (44)
			H⟶L (21)
	397	1.02	H-10⟶L (13)
			H-7⟶L (25)
			H-1⟶L+1 (12)
			H⟶L (12)
**6**	452	2.77	H-1⟶L (54)
			H⟶L+1 (37)
	431	2.53	H-1⟶L+1 (63)
			H⟶L (24)

**7**	714	0.04	S-1 ⟶S (95)
	507	0.20	S-2 ⟶S (28)
	502	0.08	S-2 ⟶S (67)
	483	0.05	S-7 ⟶S (16)
			S-5 ⟶S (13)
	446	0.11	S ⟶L+5 (13)
			S-1⟶L+4 (14)
			S-1⟶L+5 (11)
	403	0.10	S-4 ⟶S (37)

[a] H = HOMO; L = LUMO, S = SOMO

Compound **1** xyz

S	0.502864542	1.456987544	0.106190056
C	2.032947421	2.224476954	0.047762723
C	-0.214573202	2.979590459	-0.010009788
C	3.188552868	1.429472567	0.045533293
C	1.974101372	3.635001850	-0.028075222
C	-1.610234470	3.066649098	0.017441030
C	0.692469048	4.067430941	-0.079896405
C	3.191700419	0.025684319	0.016754368
C	4.509757213	2.129202568	0.004785380
H	2.736714491	4.211999682	-0.061550347
C	-2.485594079	1.966647382	0.028203126
C	-2.209364095	4.437091006	0.037865502
H	0.438880356	4.959688233	-0.152051994
N	2.066114276	-0.738447476	0.145796029
C	4.336463397	-0.812592235	-0.257701244
C	4.858481168	2.943344177	-1.061791899
C	5.418784863	1.960356997	1.033776455
N	-2.085379627	0.679513812	-0.155974300
C	-3.914606442	2.021464980	0.287026696
C	-2.987910539	4.875653717	-1.012308817
C	-1.979758739	5.315835584	1.085203002
C	2.466329009	-2.025575416	-0.038389657
C	3.895547738	-2.080588275	-0.297327186
H	5.212874899	-0.527544808	-0.383290623
H	4.253762070	3.075772722	-1.755538159
C	6.089375709	3.558744782	-1.105958478
H	5.200512409	1.424674936	1.762279361
C	6.657453397	2.587648016	0.984128646
C	-3.210959869	-0.084611407	-0.026927956
H	-4.422955350	2.783758864	0.448268419
C	-4.355530451	0.753468317	0.247412152
H	-3.166037162	4.302132024	-1.723477726
C	-3.508548418	6.157919264	-1.022485422
H	-1.458415162	5.043872129	1.806541933
C	-2.512993236	6.588202571	1.074776159
C	1.591503215	-3.125197509	-0.027338548
H	4.404216303	-2.842508559	-0.458279180
H	6.313794014	4.098279489	-1.829870924
C	6.984579045	3.378225793	-0.083662892
H	7.265258546	2.471090346	1.678433943
C	-3.207284124	-1.488020978	-0.055430811
H	-5.231613945	0.468795113	0.373279862
C	-3.265386700	7.011678587	0.014349113

H	-4.026231516	6.441061900	-1.740390228
H	-2.360289506	7.161098098	1.791590520
C	0.195841947	-3.038138869	0.000112270
C	2.190434538	-4.495661082	-0.047760319
H	7.816060973	3.792986664	-0.117176144
C	-2.051672776	-2.283018788	-0.057655558
C	-4.528688937	-2.187761062	-0.014672377
H	-3.610786965	7.874412204	0.000304047
C	-0.711400772	-4.125989435	0.070009408
N	-0.721533922	-2.015138588	-0.031493765
C	2.968780514	-4.934233875	1.002424521
C	1.960628995	-5.374410113	-1.095095558
C	-1.993033096	-3.693560344	0.018188225
C	-4.877619262	-3.001919330	1.051910741
C	-5.437914607	-2.018931526	-1.043669011
H	-0.458010382	-5.018268392	0.142167698
H	-0.450497544	-1.199567351	-0.034206497
H	3.147306180	-4.361098643	1.713355706
C	3.489823056	-6.216884937	1.012376345
H	1.439682012	-5.102827167	-1.816656133
C	2.494254186	-6.647164185	-1.084895042
H	-2.755838615	-4.270573265	0.051670734
H	-4.272495500	-3.134733389	1.745432219
C	-6.108109139	-3.617705449	1.095852538
H	-5.219245559	-1.483629973	-1.772393560
C	-6.676186547	-2.646603054	-0.994242846
C	3.246987177	-7.070258455	-0.024172039
H	4.007834161	-6.499653349	1.730559482
H	2.341889983	-7.219677965	-1.801413446
H	-6.332199438	-4.156865933	1.820043315
C	-7.002978568	-3.436805661	0.073839966
H	-7.283655901	-2.529681796	-1.688264689
H	3.592181073	-7.933008731	-0.010121135
H	-7.834658797	-3.851588196	0.107355918

Compound **2** xyz

C	−1.30798	7.64991	9.25689
C	−2.63767	7.70347	9.75518
C	−0.57922	8.70016	8.66832
S	−0.49553	6.12706	9.36409
H	−3.18721	8.47877	9.75166
C	−3.01451	6.49089	10.23700
H	−3.85636	6.29978	10.63241
C	−1.95644	5.54296	10.06260
C	−1.98203	4.18181	10.37689
C	−0.92852	3.27222	10.11449
C	−3.23550	3.63897	10.97277
C	−0.93265	1.87231	10.35083
N	0.28101	3.53833	9.52226
H	−1.63515	1.38314	10.76301
C	0.23792	1.34427	9.88889
H	0.48904	0.42889	9.92875
C	1.01780	2.39973	9.33666
C	2.27001	2.26220	8.70626
C	3.03104	3.29514	8.14901
C	2.83537	0.86895	8.60684
C	4.30100	3.16199	7.51747
N	2.73078	4.64483	8.10284
H	4.77495	2.34844	7.39345
C	4.71884	4.38894	7.12250
H	5.53845	4.57649	6.67964
C	3.73177	5.35229	7.47884
C	3.77133	6.73053	7.22307
C	2.73264	7.65387	7.49895
C	5.00362	7.27545	6.56387
C	2.76196	9.06128	7.27678
N	1.47470	7.38297	8.02970
H	3.49592	9.54610	6.91804
C	1.57584	9.60016	7.65895
H	1.34681	10.52106	7.61342
C	0.72686	8.55346	8.14215
C	−1.20978	10.03797	8.58604
C	−3.26502	3.12740	12.25688
C	−4.42236	3.63022	10.23929
H	−2.46576	3.10766	12.77055
C	−4.44924	2.63916	12.81026
H	−4.45346	2.31077	13.70164
C	−5.60390	2.63041	12.07928
H	−6.40495	2.27901	12.45115

C	−5.59614	3.14032	10.78717
H	−6.39762	3.15288	10.27767
H	−4.42578	3.96570	9.35061
C	3.64169	0.35029	9.60158
C	2.54714	0.07168	7.52707
H	3.85807	0.88554	10.35593
C	4.13711	−0.94126	9.51312
H	4.68929	−1.28228	10.20756
C	3.83791	−1.72749	8.43564
H	4.17354	−2.61551	8.38048
C	3.04975	−1.22320	7.43770
H	2.84423	−1.75937	6.68051
H	1.99718	0.40924	6.83027
C	6.22879	7.29462	7.22855
C	4.94757	7.79390	5.27542
H	6.29140	6.93474	8.10563
C	7.35690	7.83037	6.62878
H	8.18337	7.83798	7.09772
C	7.28700	8.35195	5.35702
H	8.05884	8.72635	4.94893
C	6.07676	8.32569	4.67610
H	6.02373	8.67457	3.79405
H	4.12460	7.78321	4.80054
C	−1.60148	10.72042	9.74832
C	−1.45563	10.65436	7.35884
H	−1.43929	10.32877	10.59851
C	−2.21734	11.94883	9.67426
H	−2.47710	12.39214	10.47330
C	−2.46289	12.54668	8.45141
H	−2.89356	13.39225	8.40544
C	−2.07066	11.89153	7.29233
H	−2.22581	12.29583	6.44668
H	−1.19684	10.21747	6.55619
H	1.99695	5.00329	8.42928

Compound **3** xyz

S	-0.102488450	1.805531432	-0.427933192
C	-1.371240693	2.762767857	0.135637141
C	1.065115928	2.866975417	0.134501267
C	-0.891576534	3.976050260	0.744504482
C	-2.692680601	2.331047120	-0.082151930
C	0.473358704	4.002086784	0.766297474
C	2.425243889	2.585317356	-0.081007179
H	-1.449371937	4.664528503	1.085452897
C	-2.993358165	0.961539263	-0.370808847
C	-3.832506028	3.293126455	0.021715665
H	0.977724715	4.703257403	1.161748143
C	2.852251221	1.256445332	-0.446438739
C	3.480377612	3.651472549	0.040280353
N	-2.090200668	-0.149873455	0.137443747
C	-4.085229910	0.417207385	-1.130328574
C	-3.729571078	4.591175331	-0.467707610
C	-5.001727642	2.879322300	0.649772720
N	2.112282643	0.082699154	0.043803911
C	4.012816793	0.868840994	-1.195778156
C	3.201790102	4.979695380	-0.259361948
C	4.736232910	3.296520531	0.518388994
H	-1.356225483	-0.112156549	0.621673943
C	-2.816646213	-1.321807944	-0.426315556
C	-3.956432293	-0.946581792	-1.147705844
H	-4.776379989	0.917290022	-1.547041754
H	-2.930042325	4.874008407	-0.896214657
C	-4.793390201	5.472982247	-0.326498711
C	-6.066318785	3.761378033	0.787696474
H	-5.072454251	1.992188274	0.982462963
H	1.364668911	0.045547793	0.506530508
C	2.960498581	-1.008819323	-0.454764783
H	4.630998551	1.458473058	-1.610283992
C	4.073107917	-0.503156952	-1.205516815
H	2.342420981	5.222465645	-0.586428746
C	4.177389927	5.952873590	-0.083105478
C	5.714195223	4.269001020	0.695818596
H	4.928116788	2.388459536	0.724332684
C	-2.328014864	-2.637380654	-0.154098377
H	-4.551798691	-1.545635458	-1.583370015
H	-4.722631360	6.360068838	-0.661703117
C	-5.961795830	5.057639909	0.299900918
H	-6.865028240	3.477724566	1.216726408
C	2.632743989	-2.358421277	-0.151520035

H	4.739303491	−1.027517679	−1.635487745
H	3.988086472	6.860323077	−0.288393212
C	5.434029650	5.597953058	0.394072471
H	6.572404966	4.026655685	1.022013284
C	−0.972996946	−2.926616491	0.181623801
C	−3.350757184	−3.731770978	0.001123965
H	−6.690650861	5.661268016	0.395047284
C	1.285482748	−2.784448830	0.204067202
C	3.720110690	−3.372277579	−0.048975844
H	6.102055040	6.262191651	0.515806604
N	0.119692220	−2.104271562	−0.315648738
C	−0.463089387	−3.997981316	0.924021428
C	−4.628935658	−3.444117193	0.465753244
C	−3.005403100	−5.038726685	−0.323313502
C	0.920735776	−3.919096489	0.954905805
C	4.964832812	−2.993877465	0.442607468
C	3.476029653	−4.702490949	−0.371274832
H	0.079583630	−1.377236088	−0.809958229
H	−0.984568497	−4.673546269	1.341735241
H	−4.862592807	−2.551251643	0.689632146
C	−5.563413887	−4.463511245	0.603734644
C	−3.941649315	−6.058323700	−0.185454313
H	−2.132962669	−5.235465100	−0.639802659
H	1.509369163	−4.516706149	1.397988317
H	5.132451186	−2.085382407	0.663009733
C	5.963983449	−3.945131457	0.611916145
C	4.475180147	−5.653735849	−0.201961993
H	2.625347855	−4.960730121	−0.706244549
H	−6.436812257	−4.266744261	0.921113429
C	−5.220014558	−5.770269860	0.277429694
H	−3.707182119	−6.950957718	−0.408321376
H	6.814484325	−3.686829531	0.946838646
C	5.718923955	−5.274982650	0.288792263
H	4.307387567	−6.562164982	−0.422418169
H	−5.859441753	−6.466988556	0.372295018
H	6.402113622	−5.925789993	0.404688796

Compound **4** xyz

S	−0.155420139	−1.518761386	−0.127478883
C	−1.513193393	−2.610726341	−0.099518009
C	0.981397273	−2.833150670	−0.019667883
C	−2.850219731	−2.159960405	−0.084263213
C	−1.066367781	−3.951443972	−0.003903412
C	2.374737799	−2.645013062	−0.026952126
C	0.293692142	−4.068422169	0.056795053
C	−3.205377020	−0.797886840	−0.054621021
C	−3.946200508	−3.171211309	−0.014882801
H	−1.655144814	−4.697584808	0.016119102
C	2.986143729	−1.373732323	−0.016802449
C	3.253069453	−3.850898225	−0.042214963
H	0.738184727	−4.904148638	0.143007198
N	−2.301051384	0.215889484	−0.196725844
C	−4.521695655	−0.255873609	0.253487183
C	−4.015921657	−4.082668198	1.037118179
C	−4.932125547	−3.202174644	−0.996731890
N	2.300852382	−0.216354170	0.196972410
C	4.386872182	−1.087343323	−0.301991300
C	4.165948370	−4.065550359	0.984549236
C	3.171234365	−4.792245542	−1.065522866
C	−2.986342731	1.373267637	0.017049015
C	−4.387128391	1.086847423	0.302104251
H	−5.320849671	−0.750603992	0.392809747
H	−3.344030577	−4.080697008	1.708159422
C	−5.058748206	−4.989444091	1.109630709
H	−4.895418143	−2.589200873	−1.721855946
C	−5.970473737	−4.123204882	−0.922312068
C	3.205179126	0.797416673	0.054875877
H	5.070091398	−1.721291438	−0.485970069
C	4.521430436	0.255380676	−0.253371068
H	4.243705614	−3.428447355	1.685107259
C	4.959888947	−5.195147982	0.996906916
H	2.556846411	−4.660024517	−1.777691821
C	3.976751764	−5.920709530	−1.056531289
C	−2.374585590	2.645434979	0.026893589
H	−5.070038109	1.721246828	0.486098795
H	−5.102311489	−5.601466501	1.834345449
C	−6.034204102	−5.006341009	0.132269274
H	−6.636652699	−4.142892389	−1.599255139
C	2.850370832	2.160387803	0.084196385
H	5.320898766	0.750551561	−0.392685631
H	5.577115501	−5.331328936	1.706681814

C	4.861788574	-6.130292694	-0.017570544
H	3.919177606	-6.549205366	-1.766277505
C	-0.981237161	2.833575100	0.019597891
C	-3.252963151	3.850877008	0.042472695
H	-6.749755972	-5.628663520	0.186299453
C	1.513344495	2.611153739	0.099451182
C	3.946315821	3.171187126	0.015137368
H	5.399935447	-6.913191435	-0.000167697
S	0.155571241	1.519188783	0.127412056
C	-0.293664465	4.068790104	-0.057125946
C	-4.165966600	4.065475161	-0.984563861
C	-3.171251487	4.792164864	1.065516532
C	1.066404468	3.951808941	0.003569355
C	4.015913545	4.082584553	-1.037127678
C	4.932117435	3.202090999	0.996722391
H	-0.738198763	4.904081260	-0.143017212
H	-4.243847268	3.428312696	-1.685385949
C	-4.960030601	5.195013322	-0.997185606
H	-2.556988065	4.659889857	1.777413131
C	-3.976889224	5.920582690	1.056257209
H	1.655139789	4.697514465	-0.016132281
H	3.343902127	4.080567203	-1.708436667
C	5.058619756	4.989314285	-1.109907954
H	4.895280683	2.589074034	1.721581865
C	5.970332083	4.123070222	0.922033377
H	-5.576860022	5.331253447	-1.706359269
C	-4.861542105	6.130220172	0.017896254
H	-3.918928051	6.549146145	1.766599534
H	5.102565860	5.601396493	-1.834031194
C	6.034449463	5.006273967	-0.131951855
H	6.636902877	4.142814561	1.599564783
H	-5.400471258	6.913281483	0.000452076
H	6.749229171	5.628750602	-0.186018240

Compound **5** xyz

C	-4.344745199	3.862495042	0.869253575
H	-4.482888347	3.157403776	1.490860812
C	-3.299483262	3.784627744	-0.050576981
C	-5.186363069	4.974320064	0.877912117
C	-2.361523381	2.629326789	-0.018085976
C	-3.136753023	4.810212961	-0.985745170
C	-5.005095055	5.984391153	-0.049686985
H	-5.884009517	5.037586312	1.519357283
C	-2.947089722	1.339976655	-0.087427848
C	-0.989924016	2.870243885	0.050862348
H	-2.435094832	4.762324999	-1.625392217
C	-3.995400533	5.899207146	-0.986527116
H	-5.579087424	6.740409202	-0.042191757
C	-4.262943176	0.970671519	-0.500559691
N	-2.297309330	0.167152928	0.223055029
C	-0.364809875	4.094705968	0.371770512
S	0.141158767	1.674200868	-0.266092296
H	-3.888917272	6.586984202	-1.633620818
C	-4.376205452	-0.368388517	-0.443519482
H	-4.945621583	1.572230346	-0.770959717
C	-3.131546214	-0.893111982	0.016070084
H	-1.466544899	0.109209577	0.509683601
H	-0.835853761	4.895204937	0.572775433
C	0.979020113	3.992640450	0.358801586
C	1.418453070	2.706738533	-0.005706638
H	-5.147411382	-0.874313697	-0.668904510
C	-2.742750034	-2.242684330	0.152229851
H	1.566460874	4.709447029	0.570956304
C	2.735984274	2.236612304	-0.144833944
C	-1.408645588	-2.670464874	0.226173117
C	-3.815965726	-3.293406207	0.167516896
C	3.079401416	0.876638423	-0.322814296
C	3.822707547	3.220110252	0.044631078
S	-0.149910469	-1.633176420	-0.201468042
C	-0.945452924	-3.944780316	0.604977839
C	-3.715805892	-4.382493261	-0.685453220
C	-4.941522704	-3.183394580	0.979130347
C	4.414157428	0.350634613	-0.635554391
N	2.181682915	-0.150350941	-0.199771900
C	4.839456163	2.980279730	0.961370048
C	3.851120084	4.424465251	-0.661547610
C	1.008284083	-2.831320247	0.076183004
C	0.416355057	-4.021287019	0.501604565

H	−1.505628615	−4.656060169	0.893961617
H	−2.954868141	−4.472002196	−1.248922838
C	−4.714609994	−5.341521534	−0.725242538
C	−5.920131070	−4.167403924	0.936638969
H	−5.039722845	−2.437091163	1.559011317
H	5.201775739	0.853264460	−0.806148696
C	4.291214654	−0.987273415	−0.632216825
C	2.941867668	−1.298804771	−0.330230819
H	4.858660391	2.165573779	1.449444696
C	5.836607776	3.943147806	1.160911726
C	4.845759705	5.368428362	−0.420513166
H	3.185964523	4.601702245	−1.315868560
C	2.373803741	−2.572532744	−0.133051431
H	0.910137081	−4.810040493	0.699591507
H	−4.642315819	−6.082425166	−1.316482410
C	−5.801482038	−5.218521159	0.086764557
H	−6.677653786	−4.104882358	1.507060819
H	4.985214190	−1.613033682	−0.801698404
H	6.536443964	3.765755593	1.777998690
C	5.823087515	5.123077619	0.491849385
H	4.841669037	6.189804280	−0.895800712
C	3.308375234	−3.753870897	−0.076885776
H	−6.484202821	−5.877659417	0.058322305
H	6.493014904	5.774462476	0.659653452
C	3.122328508	−4.835078717	−0.928649153
C	4.346637157	−3.778777469	0.843307257
H	2.402439621	−4.834301644	−1.548194832
C	3.999270955	−5.937260597	−0.871391041
H	4.479485702	−3.045815814	1.432881219
C	5.193595274	−4.878852862	0.901329642
C	5.013507982	−5.957943130	0.051130275
H	3.887932445	−6.664169972	−1.471972187
H	5.903770944	−4.890405416	1.532478645
H	5.591621867	−6.710252254	0.107394350

Compound **6** xyz

S	−0.029088924	1.633081293	−0.224022406
C	1.211121713	2.762137847	0.252032030
C	−1.308433140	2.717513348	0.252032266
C	2.577494476	2.496811752	0.062287249
C	0.612386944	3.945803631	0.742522332
C	−2.664493412	2.403969526	0.062287741
C	−0.751929087	3.921639871	0.742522460
C	3.533979559	3.625361110	0.054298270
C	3.078999639	1.193112293	−0.154810986
H	1.115885822	4.692908916	1.047301608
C	−3.660282149	3.497941650	0.054298944
C	−3.119458244	1.083329776	−0.154810405
H	−1.281508189	4.650448040	1.047301833
C	4.768168538	3.515504880	0.708543891
C	3.223135307	4.808152635	−0.617398428
N	2.402508835	0.044214269	0.176339758
C	4.291695568	0.776598571	−0.774351303
C	−4.889746790	3.344451003	0.708544796
C	−3.391457312	4.690999847	−0.617397808
N	−2.402647562	−0.040891120	0.176340208
C	−4.316584377	0.624135062	−0.774350496
H	4.990426777	2.719652324	1.175076481
C	5.659637293	4.573812838	0.675389021
H	2.384965280	4.895034959	−1.057480495
C	4.127156705	5.859081420	−0.643765296
H	1.614958429	0.020889221	0.568664599
C	3.130435763	−1.064452962	−0.192845695
H	4.963874988	1.354059010	−1.117720451
C	4.329244541	−0.581681698	−0.792850258
H	−5.083623288	2.541228175	1.175077425
C	−5.818072633	4.370528100	0.675390097
H	−2.556829688	4.807509529	−1.057480032
C	−4.332066617	5.709257892	−0.643764503
H	−1.614705056	−0.036312198	0.568664901
C	−3.090798548	−1.174638878	−0.192845112
H	−5.008730987	1.177431564	−1.117719517
C	−4.305951183	−0.734621919	−0.792849448
H	6.497703752	4.496166297	1.117026140
C	5.349120799	5.737534013	0.005880325
H	3.913134734	6.660864684	−1.106683098
C	2.671399721	−2.389575905	0.005585603
H	5.031481242	−1.116827326	−1.146624701
H	−6.652803826	4.263254200	1.117027373

C	−5.548900041	5.544516302	0.005881346
H	−4.146511255	6.518118175	−1.106682342
C	−2.585065896	−2.482674549	0.005586096
H	−4.988737266	−1.294298050	−1.146623762
H	5.969088023	6.456397880	−0.011268991
C	1.307870873	−2.690840789	0.200846280
C	3.653006243	−3.497666016	0.010567774
H	−6.193884433	6.240976278	−0.011267851
C	−1.211673981	−2.735465110	0.200846516
C	−3.526767738	−3.624828880	0.010568447
S	0.029663934	−1.670626377	−0.382297461
C	0.748317210	−3.835716799	0.829699322
C	3.327984099	−4.729780158	−0.565651961
C	4.911229311	−3.327449000	0.599312551
C	−0.611859471	−3.859807246	0.829699449
C	−3.158248652	−4.844659531	−0.565651353
C	−4.790159199	−3.499272842	0.599313460
H	1.278296710	−4.525011180	1.213432352
H	2.481084281	−4.851926234	−0.979923925
C	4.236887710	−5.767197244	−0.532625577
H	5.146075369	−2.495598963	0.990157768
C	5.818433259	−4.385229480	0.607890068
H	−1.117037624	−4.567435576	1.213432576
H	−2.307494716	−4.936738016	−0.979923477
C	−4.029785810	−5.913610471	−0.532624802
H	−5.054254891	−2.676259693	0.990158724
C	−5.659276667	−4.588514217	0.607891144
H	4.007407251	−6.606092288	−0.912200904
C	5.485759214	−5.595280617	0.050443209
H	6.674791448	−4.267990110	1.002556615
H	−3.770682895	−6.743852087	−0.912200175
C	−5.283901757	−5.786024914	0.050444218
H	−6.519189312	−4.501672172	1.002557851
H	6.107369565	−6.313742755	0.062352252
H	−5.879620490	−6.526047487	0.062353376

Compound **7** xyz

C	4.99220	10.22441	3.05074
C	6.34993	9.79815	3.13879
C	4.37741	10.96629	2.04332
S	3.95309	9.81633	4.46993
H	7.00946	9.96187	2.47449
C	6.57519	9.12779	4.29383
H	7.41395	8.75387	4.53530
C	5.42032	9.04839	5.09406
C	5.30899	8.45967	6.34492
C	4.14315	8.41852	7.09595
C	6.50838	7.79872	6.94575
C	3.92109	7.82114	8.36235
N	2.95849	8.87142	6.83180
H	4.58453	7.39058	8.88949
C	2.59917	7.95938	8.71196
H	2.21126	7.64821	9.52160
C	1.90481	8.63778	7.67865
C	0.54310	8.95378	7.64239
C	-0.05494	9.68158	6.63238
C	-0.29034	8.46081	8.78707
C	-1.42149	10.01278	6.52361
S	1.03645	10.20980	5.29089
H	-2.08346	9.74617	7.15124
C	-1.68487	10.76430	5.40743
H	-2.53945	11.11428	5.18074
C	-0.51434	10.94763	4.63827
C	-0.35473	11.63328	3.42108
C	0.84479	11.70272	2.69076
C	-1.55835	12.35974	2.87982
C	1.04684	12.25079	1.41142
N	2.07017	11.24988	2.96269
H	0.38128	12.69490	0.89756
C	2.32975	12.05241	1.01519
H	2.69885	12.32276	0.18333
C	3.03758	11.37227	2.05109
C	5.22272	11.39069	0.89347
C	5.83048	10.49435	0.03626
C	5.47308	12.73484	0.67075
H	5.69211	9.56265	0.16082
C	6.63731	10.92497	-0.99965
H	7.04032	10.29183	-1.58253
C	6.85606	12.26587	-1.19129
H	7.41069	12.56081	-1.90329

C	6.27691	13.17260	−0.35221
H	6.42963	14.10146	−0.47722
H	5.07350	13.37761	1.24570
C	6.58247	6.43155	6.93798
C	7.53564	8.50465	7.51808
H	5.88024	5.93137	6.53845
C	7.65208	5.76848	7.49478
H	7.68922	4.81961	7.47209
C	8.65385	6.47275	8.08006
H	9.38336	6.01816	8.48238
C	8.61051	7.84518	8.08524
H	9.31833	8.34130	8.48062
H	7.50867	9.45449	7.52259
C	−0.65172	7.14000	8.85958
C	−0.64459	9.27952	9.81779
H	−0.42671	6.56009	8.14126
C	−1.33306	6.63243	9.93951
H	−1.56078	5.71022	9.97401
C	−1.67671	7.46694	10.96765
H	−2.15777	7.12937	11.71373
C	−1.33157	8.77914	10.91585
H	−1.55984	9.35730	11.63568
H	−0.41879	10.20197	9.78457
C	−2.71250	11.71324	2.47840
C	−1.53242	13.74531	2.77623
H	−2.75804	10.76687	2.54444
C	−3.79525	12.39794	1.98635
H	−4.57302	11.92518	1.71383
C	−3.75590	13.76949	1.88794
H	−4.50083	14.25098	1.54769
C	−2.61879	14.43713	2.29453
H	−2.58656	15.38536	2.23926
H	−0.75456	14.22158	3.04222
